# Supplementary material for: Competitive Exclusion Bacterial Culture Derived from the Gut Microbiome of Nile Tilapia (Oreochromis niloticus) as a Resource to Efficiently Recover Probiotic Strains: Taxonomic, Genomic, and Functional Proof of Concept
Source: Microorganisms. 2022 Jul 8;10(7):1376. doi: 10.3390/microorganisms10071376 (PMC9321352; doi:10.3390/microorganisms10071376)
Supplement: Supplementary file 1 [file microorganisms-10-01376-s001.zip › microorganisms-1777248-supplementary.pdf]

Table S1. Evaluation of the quality of contings assembled and selection of contigs. Green: Good quality; Yellow: Accepted quality; Red: Low quality. A12 (*Lactococcus lactis*), M4 (*Priestia megaterium*), and M10 (*Priestia* sp.).

	A12				M4				M10			
**Assembly**	**Megahit**	**Skesa**	**SPAdes**	**Velvet**	**Megahit**	**Skesa**	**SPAdes**	**Velvet**	**Megahit**	**Skesa**	**SPAdes**	**Velvet**
# contigs (>= 0 bp)	398	92	91	337	1434	35	113	99	917	233	223	600
# contigs (>= 1000 bp)	56	90	48	251	52	28	25	32	173	219	103	390
# contigs (>= 5000 bp)	49	76	40	141	38	26	22	26	122	160	80	259
# contigs (>= 10000 bp)	39	61	36	79	34	24	20	23	96	125	69	164
# contigs (>= 25000 bp)	27	33	24	13	27	18	15	15	62	70	52	48
# contigs (>= 50000 bp)	14	16	13	0	17	12	10	9	37	34	35	15
Total length (>= 0 bp)	2542862	2480332	2498175	2252069	5561628	5305314	5334434	5343335	5556060	5434184	5479164	5259384
Total length (>= 1000 bp)	2484170	2479026	2488968	2217713	5317556	5300305	5313866	5328885	5436184	5424437	5452008	5191346
Total length (>= 5000 bp)	2463339	2446008	2469755	1913935	5293441	5296710	5309462	5319369	5305734	5262652	5391239	4832767
Total length (>= 10000 bp)	2389672	2343042	2440272	1456989	5261795	5278901	5291447	5293857	5122501	5008229	5312368	4132922
Total length (>= 25000 bp)	2204730	1878060	2272251	439835	5153898	5163413	5190458	5153675	4582868	4114158	5052872	2306701
Total length (>= 50000 bp)	1747674	1245119	1863988	0	4779934	4915095	4971232	4916142	3611402	2877816	4430674	1185016
# contigs	61	91	49	278	75	35	29	35	190	231	110	434
Largest contig	202730	138855	262694	47193	730183	1223276	2950775	2950484	377114	260389	441034	167341
Total length	2488200	2479969	2489652	2237376	5332033	5305314	5316929	5330689	5448399	5433377	5456842	5222642
GC (%)	34.85	34.89	34.85	35.38	37.92	37.91	37.94	37.9	37.71	37.75	37.72	37.99
N50	110147	53452	159385	13767	378794	824746	2950775	2950484	73030	52374	132222	21152
N90	20843	13794	30377	4010	49545	59281	59505	59423	15102	11660	30774	5991
L50	8	16	6	51	6	3	1	1	23	31	13	62
L90	29	52	23	164	18	10	7	7	79	115	48	236
# N's per 100 kbp	0	0	0	116.3	0	0	0	32.92	0	0	0	21.75

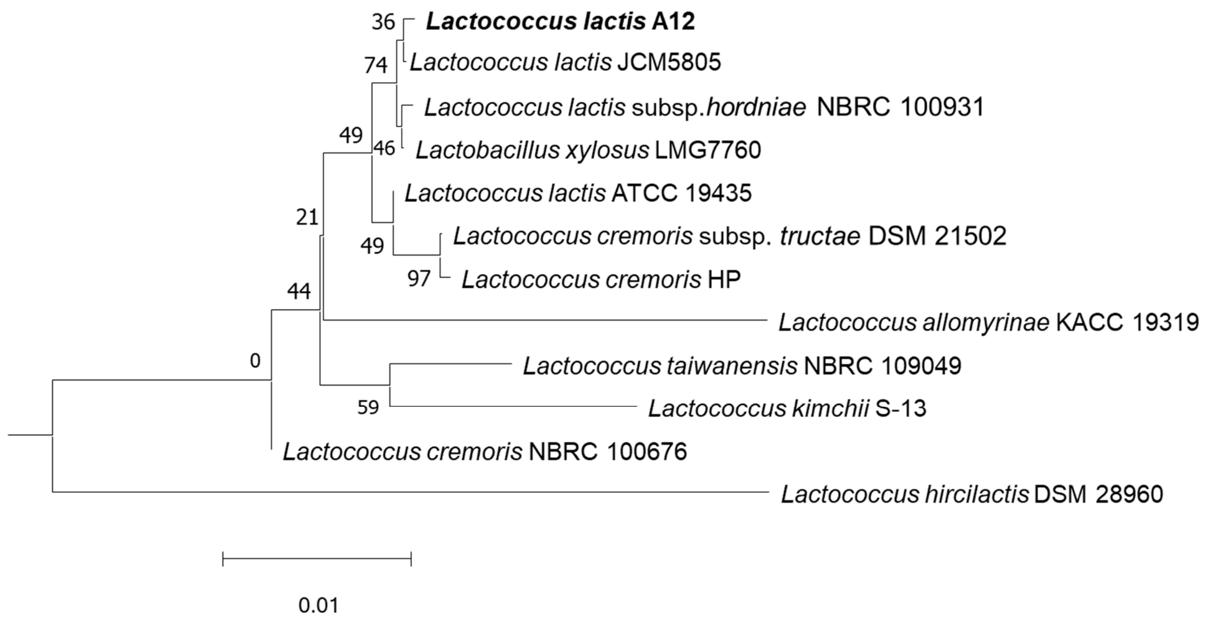
36
Lactococcus lactis A12
74
Lactococcus lactis JCM5805
Lactococcus lactis subsp.hordniae NBRC 100931
49
46
Lactobacillus xylosus LMG7760
Lactococcus lactis ATCC 19435
21
Lactococcus cremoris subsp. tructae DSM 21502
49
97
Lactococcus cremoris HP
44
Lactococcus allomyrinae KACC 19319
0
Lactococcus taiwanensis NBRC 109049
59
Lactococcus kimchii S-13
Lactococcus cremoris NBRC 100676
Lactococcus hircilactis DSM 28960
0.01

(a)

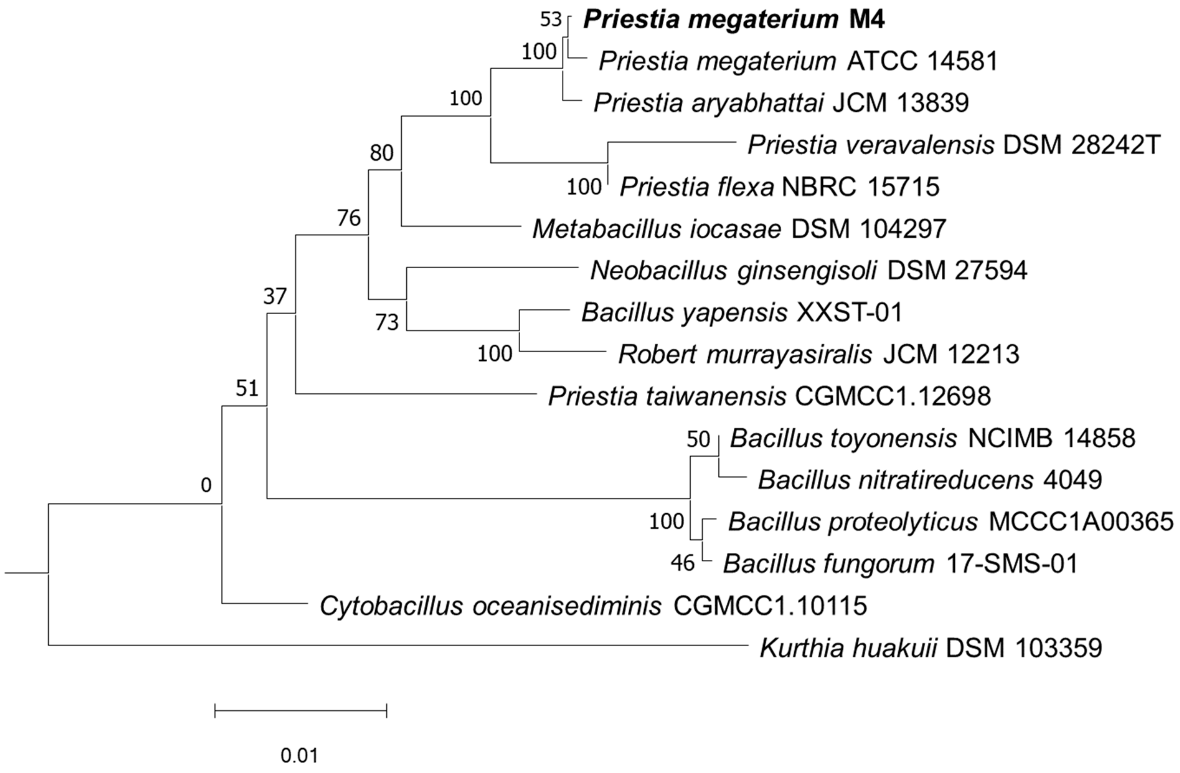
53
Priestia megaterium M4
100
Priestia megaterium ATCC 14581
100
Priestia aryabhattai JCM 13839
Priestia veravalensis DSM 28242T
80
100
Priestia flexa NBRC 15715
76
Metabacillus iocasae DSM 104297
Neobacillus ginsengisoli DSM 27594
37
73
Bacillus yapensis XXST-01
100
Robert murrayasiralis JCM 12213
51
Priestia taiwanensis CGMCC1.12698
50
Bacillus toyonensis NCIMB 14858
0
Bacillus nitratireducens 4049
100
Bacillus proteolyticus MCCC1A00365
46
Bacillus fungorum 17-SMS-01
Cytobacillus oceanisediminis CGMCC1.10115
Kurthia huakuii DSM 103359
0.01

(b)

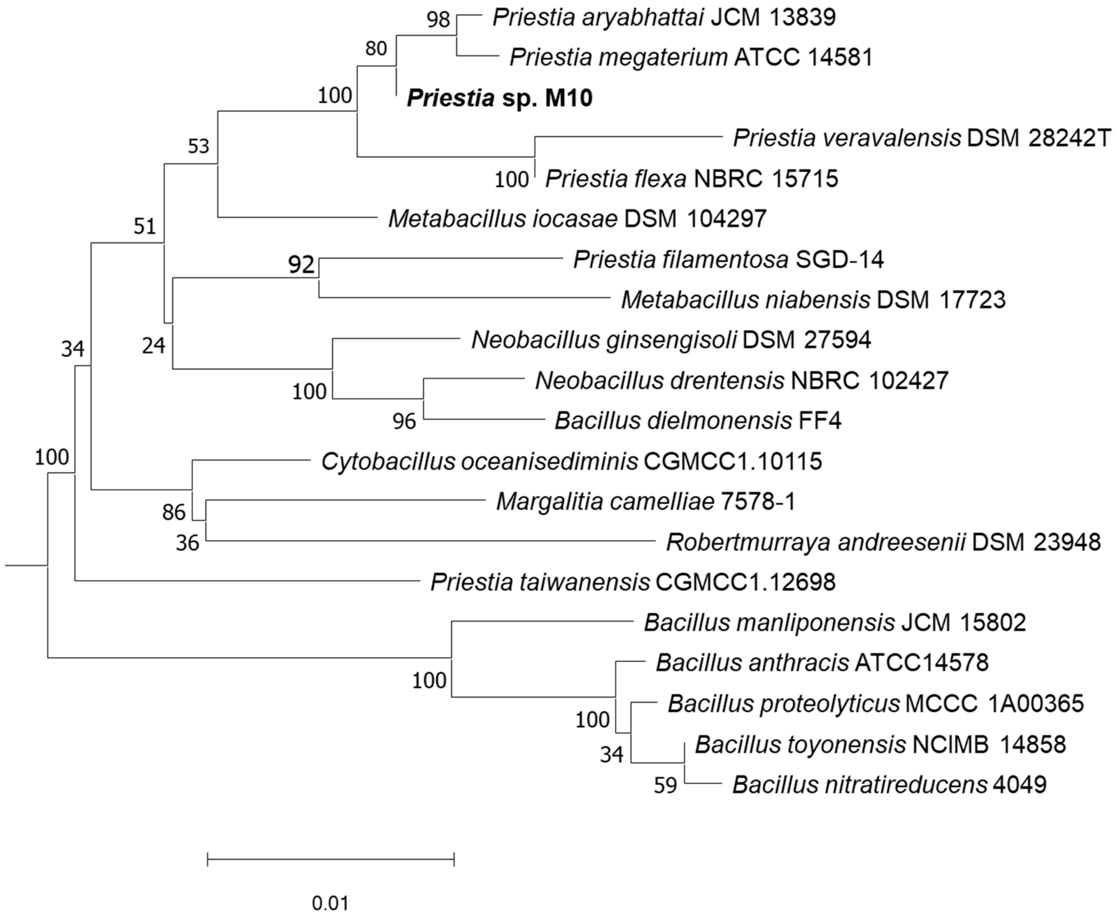

(c)

Figure S1. Tree inferred with FastME 2.1.6.1 from GBDP distances were calculated from 16S rDNA gene sequences for *Lactococcus lactis* A12 (a), *Priestia megaterium* M4 (b), and *Priestia* sp. M10 (c). The branch lengths were scaled in terms of GBDP distance formula d5. The numbers above branches were GBDP pseudo-bootstrap support values > 60 % from 100 replications, with an average branch support of 52.8 %, 74.3 %, and 73.1 % for A12, M4, and M10, respectively. The tree was rooted at the midpoint.

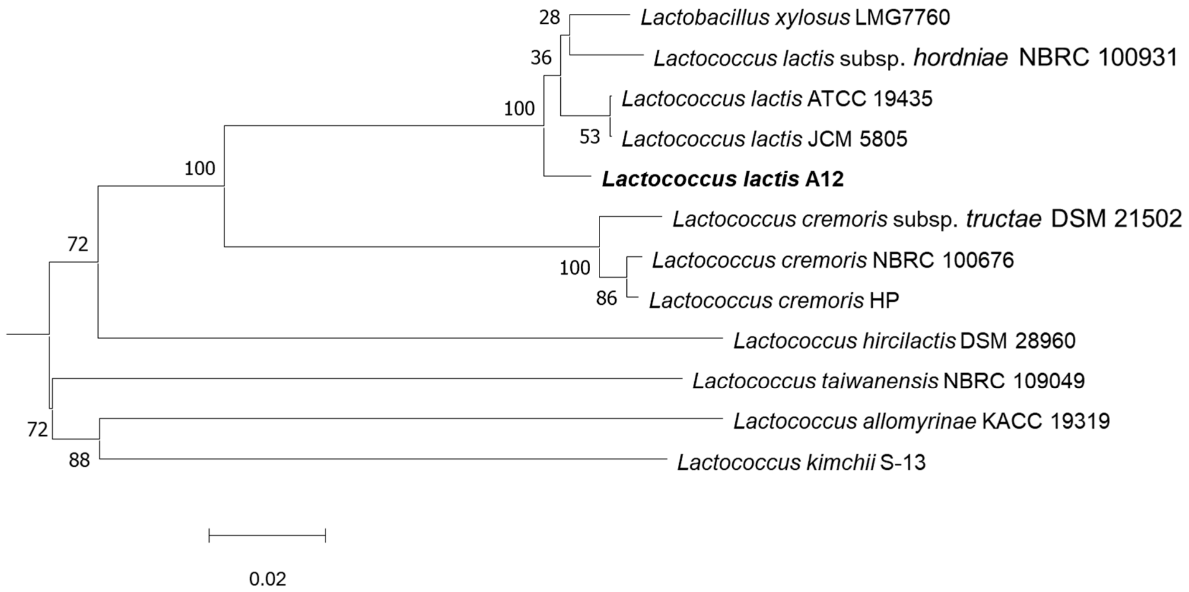
Lactobacillus xylosus LMG7760
Lactococcus lactis subsp. hordniae NBRC 100931
Lactococcus lactis ATCC 19435
Lactococcus lactis JCM 5805
Lactococcus lactis A12
Lactococcus cremoris subsp. tructae DSM 21502
Lactococcus cremoris NBRC 100676
Lactococcus cremoris HP
Lactococcus hircilactis DSM 28960
Lactococcus taiwanensis NBRC 109049
Lactococcus allomyrinae KACC 19319
Lactococcus kimchii S-13
28
36
100
53
100
72
100
86
72
88
0.02

(a)

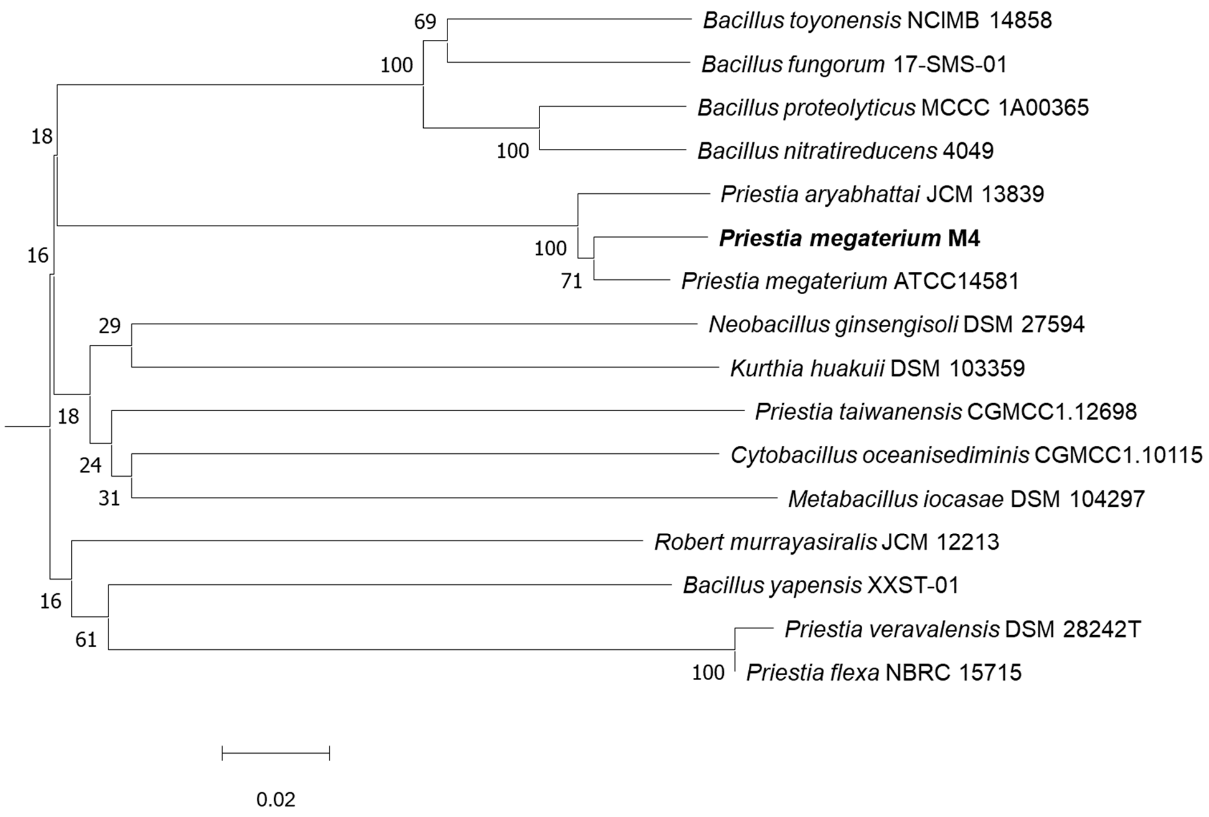

Bacillus toyonensis NCIMB 14858
Bacillus fungorum 17-SMS-01
Bacillus proteolyticus MCCC 1A00365
Bacillus nitratireducens 4049
Priestia aryabhattai JCM 13839
Priestia megaterium M4
Priestia megaterium ATCC14581
Neobacillus ginsengisoli DSM 27594
Kurthia huakuii DSM 103359
Priestia taiwanensis CGMCC1.12698
Cytobacillus oceanisediminis CGMCC1.10115
Metabacillus iocasae DSM 104297
Robert murrayasiralis JCM 12213
Bacillus yapensis XXST-01
Priestia veravalensis DSM 28242T
Priestia flexa NBRC 15715
69
100
100
18
100
16
71
29
18
24
31
16
61
100
0.02

(b)

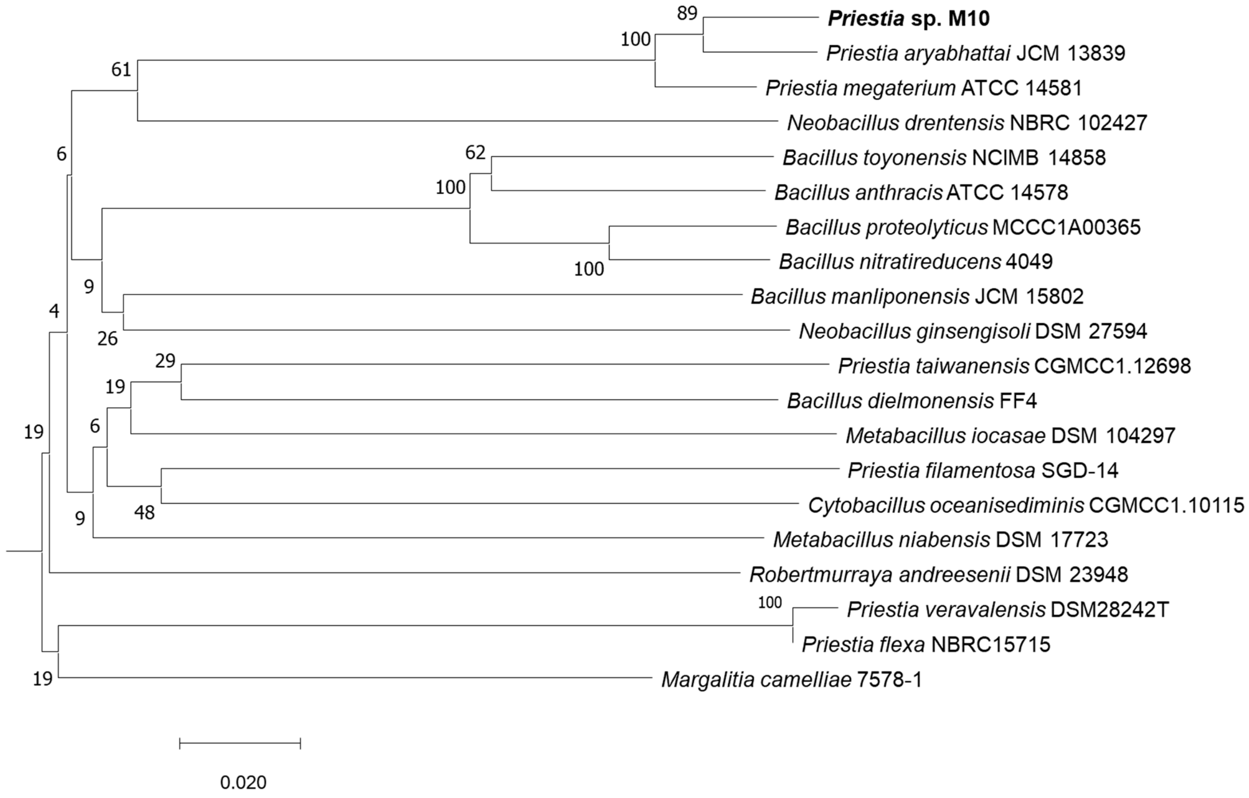

(c)

Figure S2. Tree inferred with FastME 2.1.6.1 from GBDP distances calculated from genome sequences for *Lactococcus lactis* A12 (a), *Priestia megaterium* M4 (b), and *Priestia* sp. M10 (c). The branch lengths are scaled in terms of GBDP distance formula d5. The numbers above branches are GBDP pseudo-bootstrap support values > 60 % from 100 replications, with an average branch support of 73.7 %, 56.7 %, and 46.3 %, for A12, M4, and M10, respectively. The tree was rooted at the midpoint.

Table S2. Genes related to amino acid synthesis, vitamin synthesis, carbohydrate metabolism, and adhesion identified in *Lactococcus lactis* A12.

Amino acid biosynthesis

Scaffold	Start	Stop	Length (nt)	Function	Subsystems
1	391674	393266	1593	Asparagine synthetase [glutamine-hydrolyzing] (EC 6.3.5.4)	Glutamine, Glutamate, Aspartate and Asparagine Biosynthesis
1	749473	748505	969	L-asparaginase (EC 3.5.1.1)	
1	1357512	1356697	816	Glutamate racemase (EC 5.1.1.3)	
1	1360872	1359433	1440	Glutamate synthase [NADPH] small chain (EC 1.4.1.13)	
1	1365332	1360872	4461	Glutamate synthase [NADPH] large chain (EC 1.4.1.13)	
1	1414936	1416348	1413	Transcriptional regulator, GntR family domain / Aspartate aminotransferase (EC 2.6.1.1)	
1	2009379	2008198	1182	Aspartate aminotransferase (EC 2.6.1.1)	
1	2362363	2361023	1341	Glutamine synthetase type I (EC 6.3.1.2)	
1	2380997	2380275	723	Aspartate racemase (EC 5.1.1.13)	
1	2384144	2382267	1878	Asparagine synthetase [glutamine-hydrolyzing] (EC 6.3.5.4) AsnB	
1	1210888	1211970	1083	Histidinol-phosphate aminotransferase (EC 2.6.1.9)	Histidine Biosynthesis
1	1211958	1212944	987	ATP phosphoribosyltransferase regulatory subunit (EC 2.4.2.17)	
1	1213547	1214839	1293	Histidinol dehydrogenase (EC 1.1.1.23)	
1	1215580	1216185	606	Imidazoleglycerol-phosphate dehydratase (EC 4.2.1.19)	
1	1216789	1217508	720	Phosphoribosylformimino-5-aminoimidazole carboxamide ribotide isomerase (EC 5.3.1.16)	
1	1218274	1218912	639	Phosphoribosyl-AMP cyclohydrolase (EC 3.5.4.19) / Phosphoribosyl-ATP pyrophosphatase (EC 3.6.1.31)	

1	1218913	1219722	810	Histidinol-phosphatase (EC 3.1.3.15)	
1	65621	64341	1281	O-acetylhomoserine sulfhydrylase (EC 2.5.1.49) @ O-succinylhomoserine sulfhydrylase (EC 2.5.1.48)	Methionine Biosynthesis,
1	248952	248476	477	S-ribosylhomocysteine lyase (EC 4.4.1.21) @ Autoinducer-2 production protein LuxS	
1	269251	268319	933	Cysteine synthase (EC 2.5.1.47)	
1	353092	353952	861	Methionine ABC transporter substrate-binding protein	
1	354096	354956	861	Methionine ABC transporter substrate-binding protein	
1	355074	355934	861	Methionine ABC transporter substrate-binding protein	
1	355995	356813	819	Methionine ABC transporter substrate-binding protein	
1	356917	358023	1107	Methionine ABC transporter ATP-binding protein	
1	358023	358718	696	Methionine ABC transporter permease protein	
1	358766	359314	549	Substrate-specific component MtsA of methionine-regulated ECF transporter	
1	359423	361120	1698	Duplicated ATPase component MtsB of energizing module of methionine-regulated ECF transporter	
1	361113	361934	822	Transmembrane component MtsC of energizing module of methionine-regulated ECF transporter	
1	794907	796049	1143	Cystathionine gamma-lyase (EC 4.4.1.1)	
1	796264	797184	921	Cysteine synthase (EC 2.5.1.47)	
1	1151729	1153015	1287	Homoserine dehydrogenase (EC 1.1.1.3)	
1	754452	753100	1353	Aspartokinase (EC 2.7.2.4)	Threonine and Homoserine Biosynthesis
1	1151729	1153015	1287	Homoserine dehydrogenase (EC 1.1.1.3)	
1	1153020	1153910	891	Homoserine kinase (EC 2.7.1.39)	
1	1414936	1416348	1413	Transcriptional regulator, GntR family domain / Aspartate aminotransferase (EC 2.6.1.1)	
1	1739298	1738222	1077	Aspartate-semialdehyde dehydrogenase (EC 1.2.1.11)	

1	2009379	2008198	1182	Aspartate aminotransferase (EC 2.6.1.1)	
1	2254330	2252840	1491	Threonine synthase (EC 4.2.3.1)	
1	39158	40333	1176	Aromatic amino acid aminotransferase gamma (EC 2.6.1.57) @ N-acetyl-L,L-diaminopimelate aminotransferase (EC 2.6.1.-)	Lysine Biosynthesis DAP Pathway,
1	309794	310564	771	2,3,4,5-tetrahydropyridine-2,6-dicarboxylate N-acetyltransferase (EC 2.3.1.89)	
1	310701	311855	1155	N-acetyl-L,L-diaminopimelate deacetylase (EC 3.5.1.47)	
1	754452	753100	1353	Aspartokinase (EC 2.7.2.4)	
1	1359317	1358058	1260	Diaminopimelate decarboxylase (EC 4.1.1.20)	
1	1643924	1643142	783	4-hydroxy-tetrahydrodipicolinate reductase (EC 1.17.1.8)	
1	1737412	1736519	894	4-hydroxy-tetrahydrodipicolinate synthase (EC 4.3.3.7)	
1	1739298	1738222	1077	Aspartate-semialdehyde dehydrogenase (EC 1.2.1.11)	
1	38094	36472	1623	Sulfate permease	Cysteine Biosynthesis
1	269251	268319	933	Cysteine synthase (EC 2.5.1.47)	
1	794907	796049	1143	Cystathionine gamma-lyase (EC 4.4.1.1)	
1	796264	797184	921	Cysteine synthase (EC 2.5.1.47)	
1	2032237	2031638	600	Serine acetyltransferase (EC 2.3.1.30)	
1	1221121	1222662	1542	2-isopropylmalate synthase (EC 2.3.3.13)	Branched-Chain Amino Acid Biosynthesis,
1	1222674	1223711	1038	3-isopropylmalate dehydrogenase (EC 1.1.1.85)	
1	1224003	1225385	1383	3-isopropylmalate dehydratase large subunit (EC 4.2.1.33)	
1	1225405	1225980	576	3-isopropylmalate dehydratase small subunit (EC 4.2.1.33)	
1	1226895	1228607	1713	Dihydroxy-acid dehydratase (EC 4.2.1.9)	
1	1228618	1230345	1728	Acetolactate synthase large subunit (EC 2.2.1.6)	

1	1230338	1230814	477	Acetolactate synthase small subunit (EC 2.2.1.6)	
1	1231938	1233188	1251	Threonine dehydratase biosynthetic (EC 4.3.1.19)	
1	1367167	1366145	1023	Branched-chain amino acid aminotransferase (EC 2.6.1.42)	
1	1221121	1222662	1542	2-isopropylmalate synthase (EC 2.3.3.13)	Leucine biosynthesis
1	1222674	1223711	1038	3-isopropylmalate dehydrogenase (EC 1.1.1.85)	
1	1224003	1225385	1383	3-isopropylmalate dehydratase large subunit (EC 4.2.1.33)	
1	1225405	1225980	576	3-isopropylmalate dehydratase small subunit (EC 4.2.1.33)	
1	1367167	1366145	1023	Branched-chain amino acid aminotransferase (EC 2.6.1.42)	
1	587273	588520	1248	Serine hydroxymethyltransferase (EC 2.1.2.1)	Glycine Biosynthesis,
1	860267	861370	1104	Alanine racemase (EC 5.1.1.1)	Alanine biosynthesis,
1	1367167	1366145	1023	Branched-chain amino acid aminotransferase (EC 2.6.1.42)	
1	2039615	2040589	975	Cysteine desulfurase (EC 2.8.1.7)	
1	587273	588520	1248	Serine hydroxymethyltransferase (EC 2.1.2.1)	Serine Biosynthesis
1	589412	590509	1098	Phosphoserine aminotransferase (EC 2.6.1.52)	
1	590506	591702	1197	D-3-phosphoglycerate dehydrogenase (EC 1.1.1.95)	
1	591726	592376	651	Phosphoserine phosphatase (EC 3.1.3.3)	

Vitamins Biosynthesis

Scaffold	Start	Stop	Length (nt)	Function	Subsystems
1	351135	350587	549	Substrate-specific component BioY of biotin ECF transporter	Biotin biosynthesis,
1	1073737	1073087	651	Competence protein F homolog, phosphoribosyltransferase domain; protein YhgH required for utilization of DNA as sole source of carbon and energy	
1	1652162	1651023	1140	3-ketoacyl-CoA thiolase (EC 2.3.1.16) @ Acetyl-CoA acetyltransferase (EC 2.3.1.9)	

1	1924695	1923724	972	Biotin operon repressor / Biotin--protein ligase (EC 6.3.4.9)(EC 6.3.4.10)(EC 6.3.4.11)(EC 6.3.4.15)	
1	1925270	1924701	570	Substrate-specific component BioY of biotin ECF transporter	
1	1925415	1926656	1242	Long-chain-fatty-acid--CoA ligase (EC 6.2.1.3)	
1	1926646	1927794	1149	3-ketoacyl-CoA thiolase (EC 2.3.1.16) @ Acetyl-CoA acetyltransferase (EC 2.3.1.9)	
1	352363	351815	549	Substrate-specific component ThiT of thiamin ECF transporter	Thiamin biosynthesis
1	1338605	1337958	648	Thiamin-phosphate pyrophosphorylase (EC 2.5.1.3)	
1	1340164	1339412	753	Hydroxyethylthiazole kinase (EC 2.7.1.50)	
1	1547149	1545350	1800	1-deoxy-D-xylulose 5-phosphate synthase (EC 2.2.1.7)	
1	1800764	1799022	1743	1-deoxy-D-xylulose 5-phosphate synthase (EC 2.2.1.7)	
1	2005529	2004894	636	Thiamin pyrophosphokinase (EC 2.7.6.2)	
1	738387	737275	1113	O-succinylbenzoate synthase (EC 4.2.1.113)	Menaquinone and Phylloquinone Biosynthesis,
1	739739	738384	1356	O-succinylbenzoic acid--CoA ligase (EC 6.2.1.26)	
1	741131	740289	843	Naphthoate synthase (EC 4.1.3.36)	
1	742333	741518	816	2-succinyl-6-hydroxy-2,4-cyclohexadiene-1-carboxylate synthase (EC 4.2.99.20)	
1	744005	742323	1683	2-succinyl-5-enolpyruvyl-6-hydroxy-3-cyclohexene-1-carboxylic-acid synthase (EC 2.2.1.9)	
1	745234	744059	1176	Isochorismate synthase (EC 5.4.4.2) @ Menaquinone-specific isochorismate synthase (EC 5.4.4.2)	
1	1788668	1789627	960	1,4-dihydroxy-2-naphthoate polyprenyltransferase (EC 2.5.1.74)	
1	550828	551841	1014	NAD-dependent glyceraldehyde-3-phosphate dehydrogenase (EC 1.2.1.12)	Pyridoxin (Vitamin B6) Biosynthesis
1	589412	590509	1098	Phosphoserine aminotransferase (EC 2.6.1.52)	

1	590506	591702	1197	D-3-phosphoglycerate dehydrogenase (EC 1.1.1.95)	
1	1137383	1138156	774	Hypothetical NagD-like phosphatase	
1	1547149	1545350	1800	1-deoxy-D-xylulose 5-phosphate synthase (EC 2.2.1.7)	
1	1800764	1799022	1743	1-deoxy-D-xylulose 5-phosphate synthase (EC 2.2.1.7)	
1	2429896	2428886	1011	NAD-dependent glyceraldehyde-3-phosphate dehydrogenase (EC 1.2.1.12)	
1	24246	25517	1272	tRNA(Ile)-lysidine synthetase (EC 6.3.4.19)	Folate biosynthesis cluster
1	25498	26049	552	Hypoxanthine-guanine phosphoribosyltransferase (EC 2.4.2.8)	
1	145260	145793	534	5-formyltetrahydrofolate cyclo-ligase (EC 6.3.3.2)	
1	1143812	1144318	507	Dihydrofolate reductase (EC 1.5.1.3)	
1	1146435	1146785	351	Dihydroneopterin aldolase (EC 4.1.2.25)	
1	1146864	1147913	1050	2-amino-4-hydroxy-6-hydroxymethyldihydropteridine pyrophosphokinase (EC 2.7.6.3) / GTP cyclohydrolase I (EC 3.5.4.16) type 1	
1	1147910	1148983	1074	Dihydropteroate synthase (EC 2.5.1.15)	
1	1148986	1149483	498	Dihydroneopterin triphosphate pyrophosphohydolase	
1	1149502	1150791	1290	Dihydrofolate synthase (EC 6.3.2.12) @ Folylpolyglutamate synthase (EC 6.3.2.17)	
1	1414225	1412339	1887	Para-aminobenzoate synthase, aminase component (EC 2.6.1.85) / Aminodeoxychorismate lyase (EC 4.1.3.38)	
1	1414811	1414230	582	Para-aminobenzoate synthase, amidotransferase component (EC 2.6.1.85)	
1	1596433	1595891	543	Hypoxanthine-guanine phosphoribosyltransferase (EC 2.4.2.8)	
1	1625726	1624887	840	Thymidylate synthase (EC 2.1.1.45)	

Carbohydrates

Scaffold	Start	Stop	Length (nt)	Function	Subsystems

1	678760	677804	957	Sucrose operon repressor ScrR, LacI family	Sucrose utilization
1	680175	678757	1419	Sucrose-6-phosphate hydrolase (EC 3.2.1.26)	
1	682319	683191	873	Fructokinase (EC 2.7.1.4)	
1	1555092	1554217	876	Fructokinase (EC 2.7.1.4)	
1	476733	477398	666	Beta-phosphoglucomutase (EC 5.4.2.6)	Maltose and Maltodextrin Utilization
1	695142	697145	2004	4-alpha-glucanotransferase (amylomaltase) (EC 2.4.1.25)	
1	701134	703536	2403	Glycogen phosphorylase (EC 2.4.1.1)	
1	703626	705428	1803	Neopullulanase (EC 3.2.1.135)	
1	733213	735405	2193	Maltodextrin glucosidase (EC 3.2.1.20)	
1	1301112	1300549	564	Maltose O-acetyltransferase (EC 2.3.1.79)	
1	1402686	1402060	627	Maltose O-acetyltransferase (EC 2.3.1.79)	
1	1583755	1582751	1005	Aldose 1-epimerase (EC 5.1.3.3)	
1	1804196	1803222	975	Maltose operon transcriptional repressor MalR, LacI family	
1	1806604	1804349	2256	Maltose phosphorylase (EC 2.4.1.8)	
1	1808336	1806627	1710	Alpha-glucosidase (EC 3.2.1.20)	
1	1809953	1808379	1575	Alpha-amylase (EC 3.2.1.1)	
1	1810548	1809943	606	Maltose O-acetyltransferase (EC 2.3.1.79)	
1	1814161	1812407	1755	Neopullulanase (EC 3.2.1.135)	
1	2155033	2154014	1020	Aldose 1-epimerase (EC 5.1.3.3)	
1	216882	216043	840	UDP-glucose 4-epimerase (EC 5.1.3.2)	Lactose and Galactose Uptake and Utilization
1	850298	849513	786	Galactose-1-phosphate uridylyltransferase (EC 2.7.7.10)	
1	1583755	1582751	1005	Aldose 1-epimerase (EC 5.1.3.3)	

1	2151015	2150035	981	UDP-glucose 4-epimerase (EC 5.1.3.2)	
1	2152614	2151133	1482	Galactose-1-phosphate uridylyltransferase (EC 2.7.7.10)	
1	2153987	2152788	1200	Galactokinase (EC 2.7.1.6)	
1	2155033	2154014	1020	Aldose 1-epimerase (EC 5.1.3.3)	
1	2156551	2155163	1389	Lactose and galactose permease, GPH translocator family	
1	783688	784635	948	Mannose-6-phosphate isomerase (EC 5.3.1.8)	Mannose Metabolism
1	1572505	1574658	2154	Alpha-1,2-mannosidase	
1	1836368	1836751	384	Putative regulator of the mannose operon, ManO	
1	1837632	1837991	360	Putative regulator of the mannose operon, ManO	
1	1763114	1762212	903	Ribokinase (EC 2.7.1.15)	D-ribose utilization
1	1764108	1763125	984	Ribose operon repressor	
1	1386399	1385266	1134	Endo-1,4-beta-xylanase A precursor (EC 3.2.1.8)	Xylose utilization
1	1387258	1386401	858	Possible alpha-xyloside ABC transporter, permease component	
1	1388202	1387258	945	Possible alpha-xyloside ABC transporter, permease component	
1	1578679	1577306	1374	D-xylose proton-symporter XylT	
1	1582685	1581213	1473	Xyloside transporter XynT	
1	1585263	1583758	1506	Xylulose kinase (EC 2.7.1.17)	
1	1586649	1585330	1320	Xylose isomerase (EC 5.3.1.5)	
1	1586760	1587704	945	Xylose activator XylR (AraC family)	
1	959695	960930	1236	Phosphopentomutase (EC 5.4.2.7)	Deoxyribose and Deoxynucleoside Catabolism
1	961326	962030	705	Purine nucleoside phosphorylase (EC 2.4.2.1)	
1	1497272	1496610	663	Deoxyribose-phosphate aldolase (EC 4.1.2.4)	

1	1498767	1497475	1293	Pyrimidine-nucleoside phosphorylase (EC 2.4.2.2)	
1	1763114	1762212	903	Ribokinase (EC 2.7.1.15)	
1	1386399	1385266	1134	Endo-1,4-beta-xylanase A precursor (EC 3.2.1.8)	D-Galacturonate and D-Glucuronate Utilization,
1	1743825	1743184	642	4-hydroxy-2-oxoglutarate aldolase (EC 4.1.3.16) @ 2-dehydro-3-deoxyphosphogluconate aldolase (EC 4.1.2.14)	
1	1746193	1744775	1419	Uronate isomerase (EC 5.3.1.12)	
1	1749932	1748856	1077	Mannonate dehydratase (EC 4.2.1.8)	
1	1751693	1750221	1473	D-mannonate oxidoreductase (EC 1.1.1.57)	
1	1808336	1806627	1710	Alpha-glucosidase (EC 3.2.1.20)	

Adhesion genes

Scaffold	Start	Stop	Length (nt)	Function	Subsystems
1	121388	127339	5952	Collagen adhesion protein	- none -
1	1437668	1439290	1623	Fibronectin/fibrinogen-binding protein	- none -
30	7682	4803	2880	Putative peptidoglycan bound protein (LPXTG motif) Lmo2178 homolog	- none -
1	1192997	1195465	2469	hypothetical protein	- none -
1	659325	660779	1455	Collagen adhesin	- none -
1	2267720	2267337	384	Late competence protein ComGC, access of DNA to ComEA, FIG007487	- none -
1	1136068	1136508	441	dCMP deaminase (EC 3.5.4.12) @ Late competence protein ComEB	- none -
1	1917458	1916811	648	Late competence protein ComEA, DNA receptor	- none -
1	2192829	2193494	666	Late competence protein ComC, processing protease	- none -
1	2266230	2265946	285	Late competence protein ComGG, FIG068335	- none -
1	2266652	2266269	384	Late competence protein ComGF, access of DNA to ComEA, FIG012620	- none -

1	2266974	2266678	297	Late competence protein ComGE, FIG075573	- none -
1	2267344	2266946	399	Late competence protein ComGD, access of DNA to ComEA, FIG038316	- none -
1	2267720	2267337	384	Late competence protein ComGC, access of DNA to ComEA, FIG007487	- none -
1	2268678	2267734	945	Late competence protein ComGB, access of DNA to ComEA	- none -
1	2269627	2268701	927	Late competence protein ComGA, access of DNA to ComEA	- none -
1	773853	775148	1296	Sortase A, LPXTG specific	Heme, hemin uptake and utilization systems in GramPositives,<br><br>Sortase
1	1107874	1108620	747	Sortase A, LPXTG specific	Heme, hemin uptake and utilization systems in GramPositives,<br><br>Sortase
1	2328941	2326140	2802	internalin, putative (LPXTG motif)	Listeria surface proteins: Internalin-like proteins
1	220226	222646	2421	Anchorage subunit of a-agglutinin of a-cells, highly O-glycosylated protein with N-terminal secretion signal and C-terminal signal for addition of GPI anchor to cell wall, linked to adhesion subunit Aga2p via two disulfide bonds	- none -
1	223428	224180	753	Anchorage subunit of a-agglutinin of a-cells, highly O-glycosylated protein with N-terminal secretion signal and C-terminal signal for addition of GPI anchor to cell wall, linked to adhesion subunit Aga2p via two disulfide bonds	- none -
1	658405	659298	894	Polysaccharide intercellular adhesin (PIA) biosynthesis deacetylase IcaB (EC 3.-.-.-)	Polysaccharide deacetylases
1	866208	866660	453	Anchorage subunit of a-agglutinin of a-cells, highly O-glycosylated protein with N-terminal secretion signal and C-terminal signal for addition of GPI anchor to cell wall, linked to adhesion subunit Aga2p via two disulfide bonds	- none -
1	866846	867433	588	Anchorage subunit of a-agglutinin of a-cells, highly O-glycosylated protein with N-terminal secretion signal and C-	- none -

				terminal signal for addition of GPI anchor to cell wall, linked to adhesion subunit Aga2p via two disulfide bonds	
1	868852	869640	789	Anchorage subunit of a-agglutinin of a-cells, highly O-glycosylated protein with N-terminal secretion signal and C-terminal signal for addition of GPI anchor to cell wall, linked to adhesion subunit Aga2p via two disulfide bonds	- none -
1	1629375	1626946	2430	Anchorage subunit of a-agglutinin of a-cells, highly O-glycosylated protein with N-terminal secretion signal and C-terminal signal for addition of GPI anchor to cell wall, linked to adhesion subunit Aga2p via two disulfide bonds	- none -
1	771944	773788	1845	Streptococcal hemagglutinin protein	- none -
30	4721	3228	1494	hypothetical protein	- none -
1	1484349	1485107	759	Conserved Membrane Protein (Archaea)	- none -
1	2042232	2041045	1188	Translation elongation factor Tu	Mycobacterium virulence operon involved in protein synthesis (SSU ribosomal proteins),<br><br>Translation elongation factors bacterial
1	431926	433554	1629	Heat shock protein 60 kDa family chaperone GroEL	- none -

Table S3. Genes related to amino acid synthesis, vitamin synthesis, carbohydrate metabolism, and adhesion identified in *Priestia megaterium* M4.

**Amino acid**					
**Scaffold**	**Start**	**Stop**	**Length (nt)**	**Function**	**Subsystems**
1	585134	585532	399	glutamine synthetase family protein	Glutamine, Glutamate, Aspartate and Asparagine Biosynthesis
1	612781	614628	1848	Asparagine synthetase [glutamine-hydrolyzing] (EC 6.3.5.4) YisO	
1	770815	771804	990	Glutaminase (EC 3.5.1.2)	
1	817728	819125	1398	Transcriptional regulator, GntR family domain / Aspartate aminotransferase (EC 2.6.1.1)	
1	1053289	1055226	1938	Asparagine synthetase [glutamine-hydrolyzing] (EC 6.3.5.4)	
1	1120156	1121736	1581	Ferredoxin-dependent glutamate synthase (EC 1.4.7.1)	
1	1374949	1376133	1185	Aspartate aminotransferase (EC 2.6.1.1)	
1	2057729	2062282	4554	Glutamate synthase [NADPH] large chain (EC 1.4.1.13)	
1	2062299	2063780	1482	Glutamate synthase [NADPH] small chain (EC 1.4.1.13)	
1	2157889	2156447	1443	Transcriptional regulator, GntR family domain / Aspartate aminotransferase (EC 2.6.1.1)	
1	2387073	2387315	243	Transcriptional regulator, GntR family domain / Aspartate aminotransferase (EC 2.6.1.1)	

1	2393419	2394711	1293	NAD-specific glutamate dehydrogenase (EC 1.4.1.2)	
1	2679882	2678449	1434	Transcriptional regulator, GntR family domain / Aspartate aminotransferase (EC 2.6.1.1)	
1	3070449	3071825	1377	Transcriptional regulator, GntR family domain / Aspartate aminotransferase (EC 2.6.1.1)	
1	3158531	3157101	1431	Aspartate ammonia-lyase (EC 4.3.1.1)	
1	34287	34003	285	Transition state regulatory protein AbrB	Poly-gamma-glutamate biosynthesis
1	965535	966716	1182	Poly-gamma-glutamate synthase subunit PgsB/CapB (EC 6.3.2.-)	
1	966733	967182	450	Poly-gamma-glutamate synthase subunit PgsC/CapC (EC 6.3.2.-)	
1	967223	968419	1197	Poly-gamma-glutamate synthase subunit PgsA/CapA (EC 6.3.2.-)	
1	968449	970011	1563	Gamma-glutamyltranspeptidase PgsD/CapD (EC 2.3.2.2), catalyses PGA anchorage to peptidoglycan	
1	970359	971921	1563	Gamma-glutamyltranspeptidase PgsD/CapD (EC 2.3.2.2), catalyses PGA anchorage to peptidoglycan	
1	1509190	1510878	1689	Gamma-glutamyltranspeptidase (EC 2.3.2.2) @ Glutathione hydrolase (EC 3.4.19.13)	
1	1966417	1966713	297	Transition state regulatory protein AbrB	
1	3098940	3097330	1611	Gamma-glutamyltranspeptidase (EC 2.3.2.2) @ Glutathione hydrolase (EC 3.4.19.13)	

1	3329159	3327549	1611	Gamma-glutamyltranspeptidase (EC 2.3.2.2) @ Glutathione hydrolase (EC 3.4.19.13)	
1	3569970	3570287	318	Transition state regulatory protein AbrB	
1	3877865	3875787	2079	Gamma-glutamyltranspeptidase (EC 2.3.2.2) @ Glutathione hydrolase (EC 3.4.19.13)	
1	4684072	4683278	795	Glutamate racemase (EC 5.1.1.3)	
1	1073812	1074603	792	Histidinol-phosphatase (EC 3.1.3.15)	Histidine Biosynthesis
1	3369023	3369841	819	Histidinol-phosphatase (EC 3.1.3.15)	
1	4318171	4317068	1104	Biosynthetic Aromatic amino acid aminotransferase beta (EC 2.6.1.57) @ Histidinol-phosphate aminotransferase (EC 2.6.1.9)	
1	4786897	4787691	795	Histidinol-phosphatase (EC 3.1.3.15)	
1	5014297	5013674	624	Phosphoribosyl-AMP cyclohydrolase (EC 3.5.4.19) / Phosphoribosyl-ATP pyrophosphatase (EC 3.6.1.31)	
1	5015786	5015052	735	Phosphoribosylformimino-5-aminoimidazole carboxamide ribotide isomerase (EC 5.3.1.16)	
1	5017005	5016421	585	Imidazoleglycerol-phosphate dehydratase (EC 4.2.1.19)	
1	5018349	5017081	1269	Histidinol dehydrogenase (EC 1.1.1.23)	
1	5020178	5018997	1182	ATP phosphoribosyltransferase regulatory subunit (EC 2.4.2.17)	
1	69166	70104	939	Cysteine synthase (EC 2.5.1.47)	Methionine Biosynthesis

1	98672	99331	660	Serine acetyltransferase (EC 2.3.1.30)	
1	788584	787277	1308	O-acetylhomoserine sulfhydrylase (EC 2.5.1.49) @ O-succinylhomoserine sulfhydrylase (EC 2.5.1.48)	
1	819260	820195	936	Homocysteine S-methyltransferase (EC 2.1.1.10)	
1	1275820	1272377	3444	5-methyltetrahydrofolate--homocysteine methyltransferase (EC 2.1.1.13)	
1	1277659	1275821	1839	5,10-methylenetetrahydrofolate reductase (EC 1.5.1.20) / Homolog of homocysteine-binding domain	
1	1391502	1392647	1146	Cystathionine gamma-synthase (EC 2.5.1.48)	
1	1392631	1393812	1182	Cystathionine beta-lyase (EC 4.4.1.8)	
1	1856096	1857310	1215	Cystathionine gamma-synthase (EC 2.5.1.48)	
1	1936565	1935654	912	Homoserine O-succinyltransferase (EC 2.3.1.46)	
1	2186857	2188167	1311	Methionine transporter MetT	
1	3517977	3515683	2295	5-methyltetrahydropteroyltriglutamate--homocysteine methyltransferase (EC 2.1.1.14)	
1	3821716	3820838	879	5'-methylthioadenosine nucleosidase (EC 3.2.2.16) @ S-adenosylhomocysteine nucleosidase (EC 3.2.2.9)	
1	4569687	4568554	1134	Cystathionine gamma-lyase (EC 4.4.1.1) @ Homocysteine desulfhydrase (EC 4.4.1.2)	
1	4570612	4569689	924	Cystathionine beta-synthase (EC 4.2.1.22)	
1	1296159	1294996	1164	N-acetyl-L,L-diaminopimelate aminotransferase (EC 2.6.1.-)	Lysine Biosynthesis DAP Pathway,
1	1310867	1311586	720	2,3,4,5-tetrahydropyridine-2,6-dicarboxylate N-acetyltransferase (EC 2.3.1.89)	
1	1311730	1312857	1128	N-acetyl-L,L-diaminopimelate deacetylase (EC 3.5.1.47)	

1	1362474	1363274	801	4-hydroxy-tetrahydrodipicolinate reductase (EC 1.17.1.8)	
1	2099270	2100652	1383	Aspartokinase (EC 2.7.2.4)	
1	2310107	2308779	1329	Diaminopimelate decarboxylase (EC 4.1.1.20)	
1	4138104	4137232	873	4-hydroxy-tetrahydrodipicolinate synthase (EC 4.3.3.7)	
1	4139412	4138180	1233	Aspartokinase (EC 2.7.2.4)	
1	4140495	4139452	1044	Aspartate-semialdehyde dehydrogenase (EC 1.2.1.11)	
1	4379119	4377800	1320	Diaminopimelate decarboxylase (EC 4.1.1.20)	
1	4703432	4702200	1233	Aspartokinase (EC 2.7.2.4)	
1	4923229	4922375	855	Diaminopimelate epimerase (EC 5.1.1.7)	
1	817728	819125	1398	Transcriptional regulator, GntR family domain / Aspartate aminotransferase (EC 2.6.1.1)	Threonine and Homoserine Biosynthesis
1	1374949	1376133	1185	Aspartate aminotransferase (EC 2.6.1.1)	
1	2099270	2100652	1383	Aspartokinase (EC 2.7.2.4)	
1	2157889	2156447	1443	Transcriptional regulator, GntR family domain / Aspartate aminotransferase (EC 2.6.1.1)	
1	2387073	2387315	243	Transcriptional regulator, GntR family domain / Aspartate aminotransferase (EC 2.6.1.1)	

1	2679882	2678449	1434	Transcriptional regulator, GntR family domain / Aspartate aminotransferase (EC 2.6.1.1)	
1	3070449	3071825	1377	Transcriptional regulator, GntR family domain / Aspartate aminotransferase (EC 2.6.1.1)	
1	3932310	3930901	1410	Transcriptional regulator, GntR family domain / Aspartate aminotransferase (EC 2.6.1.1)	
1	4139412	4138180	1233	Aspartokinase (EC 2.7.2.4)	
1	4140495	4139452	1044	Aspartate-semialdehyde dehydrogenase (EC 1.2.1.11)	
1	4703432	4702200	1233	Aspartokinase (EC 2.7.2.4)	
1	4927015	4926101	915	Homoserine kinase (EC 2.7.1.39)	
1	4928073	4927009	1065	Threonine synthase (EC 4.2.3.1)	
1	4929371	4928070	1302	Homoserine dehydrogenase (EC 1.1.1.3)	
1	69166	70104	939	Cysteine synthase (EC 2.5.1.47)	Cysteine Biosynthesis,
1	98672	99331	660	Serine acetyltransferase (EC 2.3.1.30)	
1	2083788	2084852	1065	Sulfate and thiosulfate binding protein CysP	
1	2084947	2085783	837	Sulfate transport system permease protein CysT	
1	2085796	2086662	867	Sulfate transport system permease protein CysW	

1	2086682	2087755	1074	Sulfate and thiosulfate import ATP-binding protein CysA (EC 3.6.3.25)	
1	2558449	2560068	1620	Sulfate permease	
1	3176578	3174857	1722	Sulfite reductase [NADPH] hemoprotein beta-component (EC 1.8.1.2)	
1	3178412	3176601	1812	Sulfite reductase [NADPH] flavoprotein alpha-component (EC 1.8.1.2)	
1	3430222	3428960	1263	Sulfate permease	
1	3463738	3462308	1431	Sulfate permease	
1	3987976	3986528	1449	Sulfate permease	
1	4569687	4568554	1134	Cystathionine gamma-lyase (EC 4.4.1.1) @ Homocysteine desulfhydrase (EC 4.4.1.2)	
1	4570612	4569689	924	Cystathionine beta-synthase (EC 4.2.1.22)	
1	4820972	4821904	933	Cysteine synthase (EC 2.5.1.47)	
1	1566728	1566306	423	3-isopropylmalate dehydrogenase (EC 1.1.1.85)	Branched-Chain Amino Acid Biosynthesis,
1	2352604	2353914	1311	Valine--pyruvate aminotransferase (EC 2.6.1.66)	
1	2415384	2416343	960	Threonine dehydratase, catabolic (EC 4.3.1.19) @ L-serine dehydratase, (PLP)-dependent (EC 4.3.1.17)	
1	2482633	2480957	1677	Dihydroxy-acid dehydratase (EC 4.2.1.9)	
1	2487512	2486439	1074	Branched-chain amino acid aminotransferase (EC 2.6.1.42)	
1	3133446	3134213	768	2-isopropylmalate synthase (EC 2.3.3.13)	

1	4018516	4017245	1272	Threonine dehydratase biosynthetic (EC 4.3.1.19)	
1	4671757	4671167	591	3-isopropylmalate dehydratase small subunit (EC 4.2.1.33)	
1	4673187	4671772	1416	3-isopropylmalate dehydratase large subunit (EC 4.2.1.33)	
1	4674355	4673246	1110	3-isopropylmalate dehydrogenase (EC 1.1.1.85)	
1	4675910	4674375	1536	2-isopropylmalate synthase (EC 2.3.3.13)	
1	4677499	4676987	513	Acetolactate synthase small subunit (EC 2.2.1.6)	
1	4679202	4677496	1707	Acetolactate synthase large subunit (EC 2.2.1.6)	
1	1566728	1566306	423	3-isopropylmalate dehydrogenase (EC 1.1.1.85)	Leucine Biosynthesis
1	2487512	2486439	1074	Branched-chain amino acid aminotransferase (EC 2.6.1.42)	
1	3133446	3134213	768	2-isopropylmalate synthase (EC 2.3.3.13)	
1	4671757	4671167	591	3-isopropylmalate dehydratase small subunit (EC 4.2.1.33)	
1	4673187	4671772	1416	3-isopropylmalate dehydratase large subunit (EC 4.2.1.33)	
1	4674355	4673246	1110	3-isopropylmalate dehydrogenase (EC 1.1.1.85)	
1	4675910	4674375	1536	2-isopropylmalate synthase (EC 2.3.3.13)	
1	1517379	1518569	1191	2-amino-3-ketobutyrate coenzyme A ligase (EC 2.3.1.29)	Glycine Biosynthesis,

1	1518607	1519566	960	L-threonine 3-dehydrogenase (EC 1.1.1.103)	
1	5111151	5109907	1245	Serine hydroxymethyltransferase (EC 2.1.2.1)	
1	186872	188062	1191	Alanine racemase (EC 5.1.1.1)	Alanine biosynthesis,
1	2352604	2353914	1311	Valine--pyruvate aminotransferase (EC 2.6.1.66)	
1	2487512	2486439	1074	Branched-chain amino acid aminotransferase (EC 2.6.1.42)	
1	4593191	4592049	1143	Cysteine desulfurase (EC 2.8.1.7)	
1	4593631	4593215	417	Iron-sulfur cluster regulator IscR	
1	4629287	4630423	1137	Cysteine desulfurase (EC 2.8.1.7)	
1	321688	322620	933	D-3-phosphoglycerate dehydrogenase (EC 1.1.1.95)	Serine Biosynthesis
1	541792	540695	1098	Phosphoserine aminotransferase (EC 2.6.1.52)	
1	1819782	1820732	951	D-3-phosphoglycerate dehydrogenase (EC 1.1.1.95)	
1	2033009	2033974	966	D-3-phosphoglycerate dehydrogenase (EC 1.1.1.95)	
1	2379804	2381003	1200	D-3-phosphoglycerate dehydrogenase (EC 1.1.1.95)	
1	3508886	3507921	966	D-3-phosphoglycerate dehydrogenase (EC 1.1.1.95)	

Vitamins

Contig	Start	Stop	Length (nt)	Function	Subsystems
1	421127	422122	996	Biotin synthase (EC 2.8.1.6)	Biotin biosynthesis,
1	499745	499137	609	Substrate-specific component BioY of biotin ECF transporter	
1	569931	571463	1533	Long-chain-fatty-acid--CoA ligase (EC 6.2.1.3)	
1	800176	800739	564	Substrate-specific component BioY of biotin ECF transporter	

1	801476	802897	1422	Adenosylmethionine-8-amino-7-oxononanoate aminotransferase (EC 2.6.1.62)	
1	1366728	1367717	990	Biotin operon repressor / Biotin--protein ligase (EC 6.3.4.9)(EC 6.3.4.10)(EC 6.3.4.11)(EC 6.3.4.15)	
1	1647743	1649239	1497	Long-chain-fatty-acid--CoA ligase (EC 6.2.1.3)	
1	1768016	1769209	1194	3-ketoacyl-CoA thiolase (EC 2.3.1.16) @ Acetyl-CoA acetyltransferase (EC 2.3.1.9)	
1	2317263	2318879	1617	Long-chain-fatty-acid--CoA ligase (EC 6.2.1.3)	
1	2331267	2332457	1191	3-ketoacyl-CoA thiolase (EC 2.3.1.16) @ Acetyl-CoA acetyltransferase (EC 2.3.1.9)	
1	2407852	2409363	1512	Long-chain-fatty-acid--CoA ligase (EC 6.2.1.3)	
1	2467094	2465661	1434	Long-chain-fatty-acid--CoA ligase (EC 6.2.1.3)	
1	2467249	2467806	558	Substrate-specific component BioY of biotin ECF transporter	
1	2468049	2468915	867	3-ketoacyl-CoA thiolase (EC 2.3.1.16) @ Acetyl-CoA acetyltransferase (EC 2.3.1.9)	
1	4711580	4709889	1692	Long-chain-fatty-acid--CoA ligase (EC 6.2.1.3)	
1	509758	510885	1128	Glycine oxidase ThiO (EC 1.4.3.19)	Thiamin biosynthesis
1	510882	511085	204	Sulfur carrier protein ThiS	
1	860069	860833	765	Hydroxymethylpyrimidine ABC transporter, ATPase component	
1	860830	861573	744	Hydroxymethylpyrimidine ABC transporter, transmembrane component	
1	861570	862580	1011	Hydroxymethylpyrimidine ABC transporter, substrate-binding component	
1	2126238	2127044	807	Hydroxyethylthiazole kinase (EC 2.7.1.50)	

1	2127058	2127702	645	Thiamin-phosphate pyrophosphorylase (EC 2.5.1.3)	
1	2224570	2225931	1362	Predicted hydroxymethylpyrimidine transporter CytX	
1	2508371	2507796	576	Substrate-specific component ThiT of thiamin ECF transporter	
1	4230108	4229461	648	Thiamin pyrophosphokinase (EC 2.7.6.2)	
1	4450436	4448535	1902	1-deoxy-D-xylulose 5-phosphate synthase (EC 2.2.1.7)	
1	4847572	4848342	771	Hydroxymethylpyrimidine ABC transporter, ATPase component	
1	4848329	4849135	807	Hydroxymethylpyrimidine ABC transporter, transmembrane component	
1	1037730	1036621	1110	N-acylamino acid racemase @ O-succinylbenzoate synthase (EC 4.2.1.113)	Menaquinone and Phylloquinone Biosynthesis,
1	2229363	2230283	921	1,4-dihydroxy-2-naphthoate polyprenyltransferase (EC 2.5.1.74)	
1	4865103	4864000	1104	O-succinylbenzoate synthase (EC 4.2.1.113)	
1	4866570	4865107	1464	O-succinylbenzoic acid--CoA ligase (EC 6.2.1.26)	
1	4867471	4866653	819	Naphthoate synthase (EC 4.1.3.36)	
1	4868297	4867488	810	2-succinyl-6-hydroxy-2,4-cyclohexadiene-1-carboxylate synthase (EC 4.2.99.20)	
1	4870055	4868301	1755	2-succinyl-5-enolpyruvyl-6-hydroxy-3-cyclohexene-1-carboxylic-acid synthase (EC 2.2.1.9)	

1	4871463	4870042	1422	Isochorismate synthase (EC 5.4.4.2) @ Menaquinone-specific isochorismate synthase (EC 5.4.4.2)	
1	321688	322620	933	D-3-phosphoglycerate dehydrogenase (EC 1.1.1.95)	Pyridoxin (Vitamin B6) Biosynthesis,
1	541792	540695	1098	Phosphoserine aminotransferase (EC 2.6.1.52)	
1	1818626	1819630	1005	4-hydroxythreonine-4-phosphate dehydrogenase (EC 1.1.1.262)	
1	1819782	1820732	951	D-3-phosphoglycerate dehydrogenase (EC 1.1.1.95)	
1	2033009	2033974	966	D-3-phosphoglycerate dehydrogenase (EC 1.1.1.95)	
1	2379804	2381003	1200	D-3-phosphoglycerate dehydrogenase (EC 1.1.1.95)	
1	3508886	3507921	966	D-3-phosphoglycerate dehydrogenase (EC 1.1.1.95)	
1	4361008	4362582	1575	D-3-phosphoglycerate dehydrogenase (EC 1.1.1.95)	
1	4450436	4448535	1902	1-deoxy-D-xylulose 5-phosphate synthase (EC 2.2.1.7)	
1	5002129	5001122	1008	NAD-dependent glyceraldehyde-3-phosphate dehydrogenase (EC 1.2.1.12)	
1	63141	64517	1377	tRNA(Ile)-lysidine synthetase (EC 6.3.4.19)	Folate biosynthesis cluster,
1	64545	65084	540	Hypoxanthine-guanine phosphoribosyltransferase (EC 2.4.2.8)	
1	70202	71617	1416	Para-aminobenzoate synthase, aminase component (EC 2.6.1.85)	
1	71620	72201	582	Para-aminobenzoate synthase, amidotransferase component (EC 2.6.1.85) @ Anthranilate synthase, amidotransferase component (EC 4.1.3.27)	

1	72206	73069	864	Aminodeoxychorismate lyase (EC 4.1.3.38)	
1	73091	73450	360	Dihydroneopterin aldolase (EC 4.1.2.25)	
1	73451	73996	546	2-amino-4-hydroxy-6-hydroxymethyldihydropteridine pyrophosphokinase (EC 2.7.6.3)	
1	794242	795090	849	Dihydropteroate synthase (EC 2.5.1.15)	
1	1063681	1064325	645	GTP cyclohydrolase I (EC 3.5.4.16) type 1	
1	1368825	1369682	858	Pantoate--beta-alanine ligase (EC 6.3.2.1)	
1	1369683	1370066	384	Aspartate 1-decarboxylase (EC 4.1.1.11)	
1	2045147	2045629	483	transmembrane protein, distant homology with ydbS	
1	4019730	4019245	486	Dihydrofolate reductase (EC 1.5.1.3)	
1	4020050	4019727	324	Thymidylate synthase (EC 2.1.1.45)	
1	4021215	4020271	945	Thymidylate synthase (EC 2.1.1.45)	

Carbohydrates

Scaffold	Start	Stop	Length (nt)	Function	Subsystems
1	1135623	1136600	978	Fructokinase (EC 2.7.1.4)	Sucrose utilization
1	1537904	1536921	984	Sucrose operon repressor ScrR, LacI family	
1	1538232	1539473	1242	Sucrose permease, major facilitator superfamily	
1	1550816	1554394	3579	Sucrose-6-phosphate hydrolase (EC 3.2.1.26)	
1	2048768	2049706	939	Fructokinase (EC 2.7.1.4)	
1	3263919	3262846	1074	Sucrose operon repressor ScrR, LacI family	
1	3742361	3743590	1230	Sucrose permease, major facilitator superfamily	
1	3835284	3833806	1479	Sucrose-6-phosphate hydrolase (EC 3.2.1.26)	

1	653013	654191	1179	Galactokinase (EC 2.7.1.6)	Lactose and Galactose Uptake and Utilization
1	654222	655745	1524	Galactose-1-phosphate uridylyltransferase (EC 2.7.7.10)	
1	1000241	1001221	981	UDP-glucose 4-epimerase (EC 5.1.3.2)	
1	1243932	1243165	768	Galactose-1-phosphate uridylyltransferase (EC 2.7.7.10)	
1	1830128	1831180	1053	Aldose 1-epimerase (EC 5.1.3.3)	
1	1094556	1095563	1008	Mannose-6-phosphate isomerase (EC 5.3.1.8)	Mannose Metabolism
1	3484493	3485704	1212	D-mannose isomerase (EC 5.3.1.7)	
1	4494902	4492623	2280	Mannose-1-phosphate guanylyltransferase / Phosphomannomutase (EC 5.4.2.8)	
1	3041314	3040307	1008	2-ketogluconate utilization repressor PtxS	2-Ketogluconate Utilization
1	3043370	3042090	1281	2-ketogluconate transporter	
1	3044332	3043373	960	2-ketogluconate kinase (EC 2.7.1.13)	
1	3045072	3044329	744	Epimerase KguE	
1	891567	892445	879	Ribokinase (EC 2.7.1.15)	Deoxyribose and Deoxynucleoside Catabolism
1	2875394	2874720	675	Deoxyribose-phosphate aldolase (EC 4.1.2.4)	
1	2876717	2875416	1302	Pyrimidine-nucleoside phosphorylase (EC 2.4.2.2)	
1	2878179	2877229	951	Deoxyribonucleoside regulator DeoR (transcriptional repressor)	

1	3988898	3988194	705	Purine nucleoside phosphorylase (EC 2.4.2.1)	
1	4388145	4387315	831	Purine nucleoside phosphorylase (EC 2.4.2.1) @ N-Ribosylnicotinamide phosphorylase (EC 2.4.2.1)	
1	4389343	4388162	1182	Phosphopentomutase (EC 5.4.2.7)	
1	718352	719251	900	6-phosphogluconate dehydrogenase, decarboxylating (EC 1.1.1.44)	D-gluconate and ketogluconates metabolism,
1	719277	720818	1542	Gluconokinase (EC 2.7.1.12)	
1	722894	724222	1329	Gluconate permease	
1	810397	811182	786	Glucose 1-dehydrogenase (EC 1.1.1.47)	
1	1017878	1018663	786	Glucose 1-dehydrogenase (EC 1.1.1.47)	
1	1125126	1126484	1359	Gluconate transporter family protein	
1	1315701	1316597	897	6-phosphogluconate dehydrogenase, decarboxylating (EC 1.1.1.44)	
1	3044332	3043373	960	2-ketogluconate kinase (EC 2.7.1.13)	
1	3292088	3291195	894	6-phosphogluconate dehydrogenase, decarboxylating (EC 1.1.1.44)	
1	3379458	3378673	786	Glucose 1-dehydrogenase (EC 1.1.1.47)	
1	4049071	4047524	1548	Gluconokinase (EC 2.7.1.12)	
1	5162468	5161056	1413	6-phosphogluconate dehydrogenase, decarboxylating (EC 1.1.1.44)	

1	1135623	1136600	978	Fructokinase (EC 2.7.1.4)	Fructose utilization,
1	1286865	1287131	267	Phosphotransferase system, phosphocarrier protein HPr	
1	1287131	1288852	1722	Phosphoenolpyruvate-protein phosphotransferase of PTS system (EC 2.7.3.9)	
1	1827487	1828155	669	Transaldolase (EC 2.2.1.2)	
1	2048768	2049706	939	Fructokinase (EC 2.7.1.4)	
1	2095527	2096279	753	Transcriptional repressor of the fructose operon, DeoR family	
1	2096276	2097190	915	1-phosphofructokinase (EC 2.7.1.56)	
1	2289763	2290431	669	Transaldolase (EC 2.2.1.2)	
1	3291148	3290480	669	Transaldolase (EC 2.2.1.2)	
1	3679719	3679039	681	Transaldolase (EC 2.2.1.2)	
1	5123634	5122990	645	Transaldolase (EC 2.2.1.2)	
1	890585	891574	990	Ribose operon repressor	D-ribose utilization
1	891567	892445	879	Ribokinase (EC 2.7.1.15)	
1	2030874	2032382	1509	Xylulose kinase (EC 2.7.1.17)	Xylose utilization
1	3412795	3411104	1692	Ribulokinase (EC 2.7.1.16)	L-Arabinose utilization
1	3522117	3520738	1380	Arabinose-proton symporter	
1	3523689	3522265	1425	L-arabinose isomerase (EC 5.3.1.4)	
1	3524417	3523719	699	L-ribulose-5-phosphate 4-epimerase (EC 5.1.3.4)	
1	3526042	3524411	1632	Ribulokinase (EC 2.7.1.16)	

Adhesion and aggregation

Scaffold	Start	Stop	Length (nt)	Function	Subsystems
1	874037	872700	1338	RNA polymerase sigma-54 factor RpoN	Flagellum,

1	1069181	1069047	135	Flagellin protein FlaA
1	1070277	1069504	774	Flagellin protein FlaA
1	1993988	1994782	795	Flagellar motor rotation protein MotA
1	1994775	1995608	834	Flagellar motor rotation protein MotB
1	4169985	4169224	762	RNA polymerase sigma factor for flagellar operon
1	4174955	4174086	870	Flagellar synthesis regulator FleN
1	4176045	4174948	1098	Flagellar biosynthesis protein FlhF
1	4178081	4176045	2037	Flagellar biosynthesis protein FlhA
1	4179194	4178112	1083	Flagellar biosynthesis protein FlhB
1	4179973	4179197	777	Flagellar biosynthesis protein FliR
1	4180245	4179976	270	Flagellar biosynthesis protein FliQ
1	4180924	4180256	669	Flagellar biosynthesis protein FliP
1	4183136	4182006	1131	Flagellar motor switch protein FliN
1	4184127	4183126	1002	Flagellar motor switch protein FliM
1	4184792	4184583	210	Flagellar protein FlbD
1	4185619	4184828	792	Flagellar hook protein FlgE
1	4186073	4185645	429	Flagellar basal-body rod modification protein FlgD
1	4188407	4186083	2325	Flagellum-specific ATP synthase FliI
1	4189170	4188382	789	Flagellar assembly protein FliH
1	4190182	4189163	1020	Flagellar motor switch protein FliG
1	4191786	4190194	1593	Flagellar M-ring protein FliF
1	4192126	4191836	291	Flagellar hook-basal body complex protein FliE
1	4192588	4192139	450	Flagellar basal-body rod protein FlgC
1	4192980	4192591	390	Flagellar basal-body rod protein FlgB

1	4285627	4283711	1917	Cell division protein FtsI [Peptidoglycan synthetase] (EC 2.4.1.129) @ Sporulation peptidoglycan synthetase SpoVD	
1	4287955	4285697	2259	Cell division protein FtsI [Peptidoglycan synthetase] (EC 2.4.1.129)	
1	4522977	4521844	1134	RNA polymerase sigma factor RpoD	
1	4796913	4796302	612	Flagellar motor rotation protein MotB	
1	4797751	4796948	804	Flagellar motor rotation protein MotA	
1	5004908	5003598	1311	RNA polymerase sigma-54 factor RpoN	
1	5050265	5049864	402	Flagellar biosynthesis protein FliS	
1	5062238	5061357	882	Flagellar hook-associated protein FlgL	
1	5063764	5062241	1524	Flagellar hook-associated protein FlgK	
1	5064250	5063783	468	Flagellar biosynthesis protein FlgN	
1	5090547	5089726	822	Flagellar basal-body rod protein FlgG	
1	5091395	5090571	825	Flagellar basal-body rod protein FlgF	
1	4481948	4481511	438	Late competence protein ComGD, access of DNA to ComEA, FIG012777	- none -
1	4482246	4481935	312	Late competence protein ComGC, access of DNA to ComEA, FIG007487	- none -
1	4483307	4482261	1047	Late competence protein ComGB, access of DNA to ComEA	- none -
1	4483954	4483298	657	Late competence protein ComGA, access of DNA to ComEA	- none -
1	4484337	4484086	252	Late competence protein ComGA, access of DNA to ComEA	- none -
1	4557266	4556700	567	dCMP deaminase (EC 3.5.4.12) @ Late competence protein ComEB	- none -
1	4557998	4557363	636	Late competence protein ComEA, DNA receptor	- none -

1	4558095	4558925	831	Late competence protein ComER, proline oxidase (EC 1.5.1.2)	- none -
1	3942529	3941204	1326	Purple acid phosphatase/fibronectin domain protein	- none -
1	4247315	4249027	1713	Fibronectin/fibrinogen-binding protein	- none -
1	118718	119908	1191	Translation elongation factor Tu	Mycobacterium virulence operon involved in protein synthesis (SSU ribosomal proteins),<br><br>Translation elongation factors bacterial
1	1733079	1734296	1218	Translation elongation factor Tu	Mycobacterium virulence operon involved in protein synthesis (SSU ribosomal proteins),<br><br>Translation elongation factors bacterial
1	208000	209631	1632	Heat shock protein 60 kDa family chaperone GroEL	- none -

Table S4. Genes related to amino acid synthesis, vitamin synthesis, carbohydrate metabolism, and adhesion identified in *Priestia* sp. M10.

Amino acid

Scaffold	Start	Stop	Length (nt)	Function	Subsystems
1	600279	601073	795	Glutamate racemase (EC 5.1.1.3)	Glutamine, Glutamate, Aspartate and Asparagine Biosynthesis,
1	931927	933207	1281	NAD-specific glutamate dehydrogenase (EC 1.4.1.2)	
1	935294	934323	972	L-asparaginase (EC 3.5.1.1)	
1	1193094	1194428	1335	Glutamine synthetase type I (EC 6.3.1.2)	
1	1327699	1329108	1410	Transcriptional regulator, GntR family domain / Aspartate aminotransferase (EC 2.6.1.1)	
1	1529762	1528650	1113	L-asparaginase (EC 3.5.1.1)	
1	2028662	2029645	984	L-asparaginase I, cytoplasmic (EC 3.5.1.1)	
1	2029666	2031096	1431	Aspartate ammonia-lyase (EC 4.3.1.1)	
1	2117824	2116448	1377	Transcriptional regulator, GntR family domain / Aspartate aminotransferase (EC 2.6.1.1)	
1	2397478	2398911	1434	Transcriptional regulator, GntR family domain / Aspartate aminotransferase (EC 2.6.1.1)	
1	2689346	2688054	1293	NAD-specific glutamate dehydrogenase (EC 1.4.1.2)	
1	2696910	2695477	1434	Transcriptional regulator, GntR family domain / Aspartate aminotransferase (EC 2.6.1.1)	
1	2927100	2928542	1443	Transcriptional regulator, GntR family domain / Aspartate aminotransferase (EC 2.6.1.1)	
1	3020058	3018577	1482	Glutamate synthase [NADPH] small chain (EC 1.4.1.13)	
1	3024628	3020075	4554	Glutamate synthase [NADPH] large chain (EC 1.4.1.13)	
1	3447917	3446475	1443	Aspartate ammonia-lyase (EC 4.3.1.1)	

1	3806176	3804992	1185	Aspartate aminotransferase (EC 2.6.1.1)	
1	4058683	4057103	1581	Ferredoxin-dependent glutamate synthase (EC 1.4.7.1)	
1	4380827	4379430	1398	Transcriptional regulator, GntR family domain / Aspartate aminotransferase (EC 2.6.1.1)	
1	4427812	4426823	990	Glutaminase (EC 3.5.1.2)	
1	4582285	4580438	1848	Asparagine synthetase [glutamine-hydrolyzing] (EC 6.3.5.4) YisO	
31	10524	11315	792	Glutamate racemase (EC 5.1.1.3)	
1	600279	601073	795	Glutamate racemase (EC 5.1.1.3)	Poly-gamma-glutamate biosynthesis
1	1389877	1391955	2079	Gamma-glutamyltranspeptidase (EC 2.3.2.2) @ Glutathione hydrolase (EC 3.4.19.13)	
1	1855389	1856999	1611	Gamma-glutamyltranspeptidase (EC 2.3.2.2) @ Glutathione hydrolase (EC 3.4.19.13)	
1	2089405	2091015	1611	Gamma-glutamyltranspeptidase (EC 2.3.2.2) @ Glutathione hydrolase (EC 3.4.19.13)	
1	3115894	3115598	297	Transition state regulatory protein AbrB	
1	3662442	3660754	1689	Gamma-glutamyltranspeptidase (EC 2.3.2.2) @ Glutathione hydrolase (EC 3.4.19.13)	
1	4232088	4230526	1563	Gamma-glutamyltranspeptidase PgsD/CapD (EC 2.3.2.2), catalyses PGA anchorage to peptidoglycan	
1	4233998	4232436	1563	Gamma-glutamyltranspeptidase PgsD/CapD (EC 2.3.2.2), catalyses PGA anchorage to peptidoglycan	
1	4235224	4234028	1197	Poly-gamma-glutamate synthase subunit PgsA/CapA (EC 6.3.2.-)	
1	4235714	4235265	450	Poly-gamma-glutamate synthase subunit PgsC/CapC (EC 6.3.2.-)	

1	4236912	4235731	1182	Poly-gamma-glutamate synthase subunit PgsB/CapB (EC 6.3.2.-)	
1	5159176	5159460	285	Transition state regulatory protein AbrB	
31	10524	11315	792	Glutamate racemase (EC 5.1.1.3)	
1	235497	236678	1182	ATP phosphoribosyltransferase regulatory subunit (EC 2.4.2.17)	Histidine Biosynthesis
1	237326	238594	1269	Histidinol dehydrogenase (EC 1.1.1.23)	
1	238669	239253	585	Imidazoleglycerol-phosphate dehydratase (EC 4.2.1.19)	
1	239888	240622	735	Phosphoribosylformimino-5-aminoimidazole carboxamide ribotide isomerase (EC 5.3.1.16)	
1	241377	242000	624	Phosphoribosyl-AMP cyclohydrolase (EC 3.5.4.19) / Phosphoribosyl-ATP pyrophosphatase (EC 3.6.1.31)	
1	495892	495098	795	Histidinol-phosphatase (EC 3.1.3.15)	
1	965956	967059	1104	Biosynthetic Aromatic amino acid aminotransferase beta (EC 2.6.1.57) @ Histidinol-phosphate aminotransferase (EC 2.6.1.9)	
1	1814562	1813756	807	Histidinol-phosphatase (EC 3.1.3.15)	
1	1990522	1991832	1311	Imidazolonepropionase (EC 3.5.2.7)	
1	3597377	3596394	984	Formiminoglutamase (EC 3.5.3.8)	
1	3598641	3597352	1290	Imidazolonepropionase (EC 3.5.2.7)	
1	3600319	3598658	1662	Urocanate hydratase (EC 4.2.1.49)	
1	3601883	3600369	1515	Histidine ammonia-lyase (EC 4.3.1.3)	
1	3651885	3651421	465	Hut operon positive regulatory protein	
1	4131096	4130305	792	Histidinol-phosphatase (EC 3.1.3.15)	
1	308167	309192	1026	Methionine ABC transporter ATP-binding protein	Methionine Biosynthesis,
1	309185	309853	669	Methionine ABC transporter permease protein	

1	309871	310707	837	Methionine ABC transporter substrate-binding protein
1	329259	330560	1302	Homoserine dehydrogenase (EC 1.1.1.3)
1	331615	332529	915	Homoserine kinase (EC 2.7.1.39)
1	350542	349370	1173	Cystathionine beta-lyase (EC 4.4.1.8)
1	352219	353379	1161	Cystathionine beta-lyase (EC 4.4.1.8)
1	431334	431807	474	S-ribosylhomocysteine lyase (EC 4.4.1.21) @ Autoinducer-2 production protein LuxS
1	439998	441200	1203	S-adenosylmethionine synthetase (EC 2.5.1.6)
1	462057	461125	933	Cysteine synthase (EC 2.5.1.47)
1	712273	712914	642	SAM-dependent methlytransferase YrrT
1	712932	713627	696	5'-methylthioadenosine nucleosidase (EC 3.2.2.16) @ S-adenosylhomocysteine nucleosidase (EC 3.2.2.9)
1	713771	714694	924	Cystathionine beta-synthase (EC 4.2.1.22)
1	714696	715829	1134	Cystathionine gamma-lyase (EC 4.4.1.1) @ Homocysteine desulfhydrase (EC 4.4.1.2)
1	1442005	1442883	879	5'-methylthioadenosine nucleosidase (EC 3.2.2.16) @ S-adenosylhomocysteine nucleosidase (EC 3.2.2.9)
1	1661394	1663688	2295	5-methyltetrahydropteroyltriglutamate--homocysteine methyltransferase (EC 2.1.1.14)
1	2894223	2892913	1311	Methionine transporter MetT
1	3145696	3146607	912	Homoserine O-succinyltransferase (EC 2.3.1.46)
1	3232746	3231532	1215	Cystathionine gamma-synthase (EC 2.5.1.48)

1	3608876	3608025	852	Methionine ABC transporter substrate-binding protein	
1	3609516	3608917	600	Methionine ABC transporter permease protein	
1	3610591	3609509	1083	Methionine ABC transporter ATP-binding protein	
1	3611438	3610647	792	Methionine ABC transporter substrate-binding protein	
1	3612719	3612054	666	Methionine ABC transporter permease protein	
1	3613733	3612720	1014	Methionine ABC transporter ATP-binding protein	
1	3614564	3613746	819	Methionine ABC transporter substrate-binding protein	
1	3788629	3787448	1182	Cystathionine beta-lyase (EC 4.4.1.8)	
1	3789758	3788613	1146	Cystathionine gamma-synthase (EC 2.5.1.48)	
1	3904887	3906725	1839	5,10-methylenetetrahydrofolate reductase (EC 1.5.1.20) / Homolog of homocysteine-binding domain	
1	3906726	3910169	3444	5-methyltetrahydrofolate--homocysteine methyltransferase (EC 2.1.1.13)	
1	4379295	4378360	936	Homocysteine S-methyltransferase (EC 2.1.1.10)	
1	4410139	4411446	1308	O-acetylhomoserine sulfhydrylase (EC 2.5.1.49) @ O-succinylhomoserine sulfhydrylase (EC 2.5.1.48)	
1	5104892	5104233	660	Serine acetyltransferase (EC 2.3.1.30)	
1	5124289	5123351	939	Cysteine synthase (EC 2.5.1.47)	
1	335401	336255	855	Diaminopimelate epimerase (EC 5.1.1.7)	Lysine Biosynthesis DAP Pathway,
1	580921	582153	1233	Aspartokinase (EC 2.7.2.4)	
1	905073	906392	1320	Diaminopimelate decarboxylase (EC 4.1.1.20)	
1	1145933	1146976	1044	Aspartate-semialdehyde dehydrogenase (EC 1.2.1.11)	

1	1147016	1148248	1233	Aspartokinase (EC 2.7.2.4)	
1	1148324	1149196	873	4-hydroxy-tetrahydrodipicolinate synthase (EC 4.3.3.7)	
1	2771723	2773051	1329	Diaminopimelate decarboxylase (EC 4.1.1.20)	
1	2983518	2982136	1383	Aspartokinase (EC 2.7.2.4)	
1	3818650	3817850	801	4-hydroxy-tetrahydrodipicolinate reductase (EC 1.17.1.8)	
1	3870944	3869817	1128	N-acetyl-L,L-diaminopimelate deacetylase (EC 3.5.1.47)	
1	3871808	3871089	720	2,3,4,5-tetrahydropyridine-2,6-dicarboxylate N-acetyltransferase (EC 2.3.1.89)	
1	3886563	3887729	1167	N-acetyl-L,L-diaminopimelate aminotransferase (EC 2.6.1.-)	
1	329259	330560	1302	Homoserine dehydrogenase (EC 1.1.1.3)	Threonine and Homoserine Biosynthesis
1	330557	331621	1065	Threonine synthase (EC 4.2.3.1)	
1	331615	332529	915	Homoserine kinase (EC 2.7.1.39)	
1	580921	582153	1233	Aspartokinase (EC 2.7.2.4)	
1	1145933	1146976	1044	Aspartate-semialdehyde dehydrogenase (EC 1.2.1.11)	
1	1147016	1148248	1233	Aspartokinase (EC 2.7.2.4)	
1	1327699	1329108	1410	Transcriptional regulator, GntR family domain / Aspartate aminotransferase (EC 2.6.1.1)	
1	2117824	2116448	1377	Transcriptional regulator, GntR family domain / Aspartate aminotransferase (EC 2.6.1.1)	
1	2397478	2398911	1434	Transcriptional regulator, GntR family domain / Aspartate aminotransferase (EC 2.6.1.1)	
1	2696910	2695477	1434	Transcriptional regulator, GntR family domain / Aspartate aminotransferase (EC 2.6.1.1)	
1	2927100	2928542	1443	Transcriptional regulator, GntR family domain / Aspartate aminotransferase (EC 2.6.1.1)	

1	2983518	2982136	1383	Aspartokinase (EC 2.7.2.4)	
1	3806176	3804992	1185	Aspartate aminotransferase (EC 2.6.1.1)	
1	4380827	4379430	1398	Transcriptional regulator, GntR family domain / Aspartate aminotransferase (EC 2.6.1.1)	
1	366642	367355	714	Phosphoadenylyl-sulfate reductase [thioredoxin] (EC 1.8.4.8)	Cysteine Biosynthesis
1	369671	370270	600	Adenylylsulfate kinase (EC 2.7.1.25)	
1	462057	461125	933	Cysteine synthase (EC 2.5.1.47)	
1	713771	714694	924	Cystathionine beta-synthase (EC 4.2.1.22)	
1	714696	715829	1134	Cystathionine gamma-lyase (EC 4.4.1.1) @ Homocysteine desulfhydrase (EC 4.4.1.2)	
1	1270978	1272426	1449	Sulfate permease	
1	1761055	1762317	1263	Sulfate permease	
1	2009773	2011584	1812	Sulfite reductase [NADPH] flavoprotein alpha-component (EC 1.8.1.2)	
1	2011607	2013328	1722	Sulfite reductase [NADPH] hemoprotein beta-component (EC 1.8.1.2)	
1	2504993	2503374	1620	Sulfate permease	
1	2996108	2995035	1074	Sulfate and thiosulfate import ATP-binding protein CysA (EC 3.6.3.25)	
1	2996994	2996128	867	Sulfate transport system permease protein CysW	
1	2997843	2997007	837	Sulfate transport system permease protein CysT	
1	2999002	2997938	1065	Sulfate and thiosulfate binding protein CysP	
1	5104892	5104233	660	Serine acetyltransferase (EC 2.3.1.30)	

1	5124289	5123351	939	Cysteine synthase (EC 2.5.1.47)	
1	335401	336255	855	Diaminopimelate epimerase (EC 5.1.1.7)	Lysine Biosynthesis DAP Pathway,
1	580921	582153	1233	Aspartokinase (EC 2.7.2.4)	
1	905073	906392	1320	Diaminopimelate decarboxylase (EC 4.1.1.20)	
1	1145933	1146976	1044	Aspartate-semialdehyde dehydrogenase (EC 1.2.1.11)	
1	1147016	1148248	1233	Aspartokinase (EC 2.7.2.4)	
1	1148324	1149196	873	4-hydroxy-tetrahydrodipicolinate synthase (EC 4.3.3.7)	
1	2771723	2773051	1329	Diaminopimelate decarboxylase (EC 4.1.1.20)	
1	2983518	2982136	1383	Aspartokinase (EC 2.7.2.4)	
1	3818650	3817850	801	4-hydroxy-tetrahydrodipicolinate reductase (EC 1.17.1.8)	
1	3870944	3869817	1128	N-acetyl-L,L-diaminopimelate deacetylase (EC 3.5.1.47)	
1	3871808	3871089	720	2,3,4,5-tetrahydropyridine-2,6-dicarboxylate N-acetyltransferase (EC 2.3.1.89)	
1	3886563	3887729	1167	N-acetyl-L,L-diaminopimelate aminotransferase (EC 2.6.1.-)	
1	605156	606862	1707	Acetolactate synthase large subunit (EC 2.2.1.6)	Branched-Chain Amino Acid Biosynthesis
1	606859	607371	513	Acetolactate synthase small subunit (EC 2.2.1.6)	
1	608448	609983	1536	2-isopropylmalate synthase (EC 2.3.3.13)	
1	610003	611112	1110	3-isopropylmalate dehydrogenase (EC 1.1.1.85)	
1	611171	612586	1416	3-isopropylmalate dehydratase large subunit (EC 4.2.1.33)	
1	612601	613191	591	3-isopropylmalate dehydratase small subunit (EC 4.2.1.33)	
1	1240401	1241672	1272	Threonine dehydratase biosynthetic (EC 4.3.1.19)	
1	2054935	2054168	768	2-isopropylmalate synthase (EC 2.3.3.13)	

1	2577353	2578426	1074	Branched-chain amino acid aminotransferase (EC 2.6.1.42)	
1	2582236	2583912	1677	Dihydroxy-acid dehydratase (EC 4.2.1.9)	
1	2730741	2729440	1302	Valine--pyruvate aminotransferase (EC 2.6.1.66)	
1	3593469	3594539	1071	3-isopropylmalate dehydrogenase (EC 1.1.1.85)	
1	608448	609983	1536	2-isopropylmalate synthase (EC 2.3.3.13)	Leucine Biosynthesis
1	610003	611112	1110	3-isopropylmalate dehydrogenase (EC 1.1.1.85)	
1	611171	612586	1416	3-isopropylmalate dehydratase large subunit (EC 4.2.1.33)	
1	612601	613191	591	3-isopropylmalate dehydratase small subunit (EC 4.2.1.33)	
1	2054935	2054168	768	2-isopropylmalate synthase (EC 2.3.3.13)	
1	2577353	2578426	1074	Branched-chain amino acid aminotransferase (EC 2.6.1.42)	
1	3593469	3594539	1071	3-isopropylmalate dehydrogenase (EC 1.1.1.85)	
1	144828	146072	1245	Serine hydroxymethyltransferase (EC 2.1.2.1)	Glycine Biosynthesis,
1	3653056	3652097	960	L-threonine 3-dehydrogenase (EC 1.1.1.103)	
1	3654284	3653094	1191	2-amino-3-ketobutyrate coenzyme A ligase (EC 2.3.1.29)	
1	655082	653946	1137	Cysteine desulfurase (EC 2.8.1.7)	Alanine biosynthesis,
1	690736	691152	417	Iron-sulfur cluster regulator IscR	
1	691176	692318	1143	Cysteine desulfurase (EC 2.8.1.7)	
1	2356038	2354920	1119	Cysteine desulfurase (EC 2.8.1.7)	
1	2577353	2578426	1074	Branched-chain amino acid aminotransferase (EC 2.6.1.42)	
1	2730741	2729440	1302	Valine--pyruvate aminotransferase (EC 2.6.1.66)	
1	5017012	5015822	1191	Alanine racemase (EC 5.1.1.1)	

1	144828	146072	1245	Serine hydroxymethyltransferase (EC 2.1.2.1)	Serine Biosynthesis
1	1670039	1671004	966	D-3-phosphoglycerate dehydrogenase (EC 1.1.1.95)	
1	2704330	2703131	1200	D-3-phosphoglycerate dehydrogenase (EC 1.1.1.95)	
1	3049350	3048385	966	D-3-phosphoglycerate dehydrogenase (EC 1.1.1.95)	
1	4642166	4643263	1098	Phosphoserine aminotransferase (EC 2.6.1.52)	
1	4842116	4841184	933	D-3-phosphoglycerate dehydrogenase (EC 1.1.1.95)	

Vitamins

Scaffold	Start	Stop	Length (nt)	Function	Subsystems
1	374923	373337	1587	Long-chain-fatty-acid--CoA ligase (EC 6.2.1.3)	Biotin biosynthesis,
1	572782	574473	1692	Long-chain-fatty-acid--CoA ligase (EC 6.2.1.3)	
1	1536089	1534911	1179	8-amino-7-oxononanoate synthase (EC 2.3.1.47)	
1	2596823	2595957	867	3-ketoacyl-CoA thiolase (EC 2.3.1.16) @ Acetyl-CoA acetyltransferase (EC 2.3.1.9)	
1	2597623	2597066	558	Substrate-specific component BioY of biotin ECF transporter	
1	2597777	2599210	1434	Long-chain-fatty-acid--CoA ligase (EC 6.2.1.3)	
1	2671636	2670125	1512	Long-chain-fatty-acid--CoA ligase (EC 6.2.1.3)	
1	2750565	2749375	1191	3-ketoacyl-CoA thiolase (EC 2.3.1.16) @ Acetyl-CoA acetyltransferase (EC 2.3.1.9)	
1	2764575	2762959	1617	Long-chain-fatty-acid--CoA ligase (EC 6.2.1.3)	
1	3517402	3515906	1497	Long-chain-fatty-acid--CoA ligase (EC 6.2.1.3)	
1	3814396	3813407	990	Biotin operon repressor / Biotin--protein ligase (EC 6.3.4.9)(EC 6.3.4.10)(EC 6.3.4.11)(EC 6.3.4.15)	

1	4397158	4395785	1374	Adenosylmethionine-8-amino-7-oxononanoate aminotransferase (EC 2.6.1.62)	
1	4398456	4397893	564	Substrate-specific component BioY of biotin ECF transporter	
1	4614046	4612514	1533	Long-chain-fatty-acid--CoA ligase (EC 6.2.1.3)	
1	4684083	4684691	609	Substrate-specific component BioY of biotin ECF transporter	
1	4762038	4761043	996	Biotin synthase (EC 2.8.1.6)	
1	434741	433935	807	Hydroxymethylpyrimidine ABC transporter, transmembrane component	Thiamin biosynthesis
1	435498	434728	771	Hydroxymethylpyrimidine ABC transporter, ATPase component	
1	833784	835685	1902	1-deoxy-D-xylulose 5-phosphate synthase (EC 2.2.1.7)	
1	1053946	1054593	648	Thiamin pyrophosphokinase (EC 2.7.6.2)	
1	2556158	2556736	579	Substrate-specific component ThiT of thiamin ECF transporter	
1	2856653	2855292	1362	Predicted hydroxymethylpyrimidine transporter CytX	
1	2958173	2957529	645	Thiamin-phosphate pyrophosphorylase (EC 2.5.1.3)	
1	2958993	2958187	807	Hydroxyethylthiazole kinase (EC 2.7.1.50)	
1	4337455	4336445	1011	Hydroxymethylpyrimidine ABC transporter, substrate-binding component	
1	4338195	4337452	744	Hydroxymethylpyrimidine ABC transporter, transmembrane component	
1	4338956	4338192	765	Hydroxymethylpyrimidine ABC transporter, ATPase component	
1	4672948	4672745	204	Sulfur carrier protein ThiS	
1	4674072	4672945	1128	Glycine oxidase ThiO (EC 1.4.3.19)	

1	411591	413012	1422	Isochorismate synthase (EC 5.4.4.2) @ Menaquinone-specific isochorismate synthase (EC 5.4.4.2)	Menaquinone and Phylloquinone Biosynthesis,
1	412999	414753	1755	2-succinyl-5-enolpyruvyl-6-hydroxy-3-cyclohexene-1-carboxylic-acid synthase (EC 2.2.1.9)	
1	414757	415566	810	2-succinyl-6-hydroxy-2,4-cyclohexadiene-1-carboxylate synthase (EC 4.2.99.20)	
1	415583	416401	819	Naphthoate synthase (EC 4.1.3.36)	
1	416484	417947	1464	O-succinylbenzoic acid--CoA ligase (EC 6.2.1.26)	
1	417951	419054	1104	O-succinylbenzoate synthase (EC 4.2.1.113)	
1	2851852	2850929	924	1,4-dihydroxy-2-naphthoate polyprenyltransferase (EC 2.5.1.74)	
1	4168867	4169976	1110	N-acylamino acid racemase @ O-succinylbenzoate synthase (EC 4.2.1.113)	
1	253547	254554	1008	NAD-dependent glyceraldehyde-3-phosphate dehydrogenase (EC 1.2.1.12)	Pyridoxin (Vitamin B6) Biosynthesis
1	325424	326194	771	Hypothetical NagD-like phosphatase	
1	833784	835685	1902	1-deoxy-D-xylulose 5-phosphate synthase (EC 2.2.1.7)	
1	923118	921544	1575	D-3-phosphoglycerate dehydrogenase (EC 1.1.1.95)	
1	1670039	1671004	966	D-3-phosphoglycerate dehydrogenase (EC 1.1.1.95)	
1	2704330	2703131	1200	D-3-phosphoglycerate dehydrogenase (EC 1.1.1.95)	
1	3049350	3048385	966	D-3-phosphoglycerate dehydrogenase (EC 1.1.1.95)	
1	3273613	3272603	1011	4-hydroxythreonine-4-phosphate dehydrogenase (EC 1.1.1.262)	
1	4642166	4643263	1098	Phosphoserine aminotransferase (EC 2.6.1.52)	
1	4842116	4841184	933	D-3-phosphoglycerate dehydrogenase (EC 1.1.1.95)	
1	635480	636778	1299	Dihydrofolate synthase (EC 6.3.2.12) @ Folylpolyglutamate synthase (EC 6.3.2.17)	Folate Biosynthesis,

1	792390	792956	567	5-formyltetrahydrofolate cyclo-ligase (EC 6.3.3.2)	
1	952150	952716	567	GTP cyclohydrolase I (EC 3.5.4.16) type 1	
1	1238380	1239174	795	Thymidylate synthase (EC 2.1.1.45)	
1	1239171	1239656	486	Dihydrofolate reductase (EC 1.5.1.3)	
1	3037214	3036732	483	transmembrane protein, distant homology with ydbS	
1	3811440	3811057	384	Aspartate 1-decarboxylase (EC 4.1.1.11)	
1	3812298	3811441	858	Pantoate--beta-alanine ligase (EC 6.3.2.1)	
1	4150928	4150284	645	GTP cyclohydrolase I (EC 3.5.4.16) type 1	
1	4404695	4403847	849	Dihydropteroate synthase (EC 2.5.1.15)	
1	5120004	5119459	546	2-amino-4-hydroxy-6-hydroxymethyldihydropteridine pyrophosphokinase (EC 2.7.6.3)	
1	5120364	5120005	360	Dihydroneopterin aldolase (EC 4.1.2.25)	
1	5121249	5120386	864	Aminodeoxychorismate lyase (EC 4.1.3.38)	
1	5121835	5121254	582	Para-aminobenzoate synthase, amidotransferase component (EC 2.6.1.85) @ Anthranilate synthase, amidotransferase component (EC 4.1.3.27)	
1	5123253	5121838	1416	Para-aminobenzoate synthase, aminase component (EC 2.6.1.85)	
1	5128912	5128373	540	Hypoxanthine-guanine phosphoribosyltransferase (EC 2.4.2.8)	
1	5130316	5128940	1377	tRNA(Ile)-lysidine synthetase (EC 6.3.4.19)	

Carbohydrates

Contig	Start	Stop	Length (nt)	Function	Subsystems
1	1429499	1430977	1479	Sucrose-6-phosphate hydrolase (EC 3.2.1.26)	Sucrose utilization

1	1922219	1923292	1074	Sucrose operon repressor ScrR, LacI family	
1	3033599	3032661	939	Fructokinase (EC 2.7.1.4)	
1	3621214	3617636	3579	Sucrose-6-phosphate hydrolase (EC 3.2.1.26)	
1	3633800	3632559	1242	Sucrose permease, major facilitator superfamily	
1	3634127	3635110	984	Sucrose operon repressor ScrR, LacI family	
1	4043879	4042902	978	Fructokinase (EC 2.7.1.4)	
1	3264389	3263334	1056	Aldose 1-epimerase (EC 5.1.3.3)	Lactose and Galactose Uptake and Utilization
1	3938481	3939248	768	Galactose-1-phosphate uridylyltransferase (EC 2.7.7.10)	
1	4126003	4125053	951	UDP-glucose 4-epimerase (EC 5.1.3.2)	
1	4201829	4200849	981	UDP-glucose 4-epimerase (EC 5.1.3.2)	
1	4539177	4537654	1524	Galactose-1-phosphate uridylyltransferase (EC 2.7.7.10)	
1	4540386	4539208	1179	Galactokinase (EC 2.7.1.6)	
1	4955842	4954925	918	UDP-glucose 4-epimerase (EC 5.1.3.2)	
1	4957396	4958286	891	UDP-glucose 4-epimerase (EC 5.1.3.2)	
1	789670	791949	2280	Mannose-1-phosphate guanylyltransferase / Phosphomannomutase (EC 5.4.2.8)	Mannose Metabolism
1	1694801	1693590	1212	D-mannose isomerase (EC 5.3.1.7)	
1	3341317	3341655	339	Mannose-6-phosphate isomerase (EC 5.3.1.8)	
1	2143196	2143939	744	Epimerase KguE	2-Ketogluconate Utilization
1	2143936	2144895	960	2-ketogluconate kinase (EC 2.7.1.13)	
1	2144898	2146178	1281	2-ketogluconate transporter	

1	2146954	2147961	1008	2-ketogluconate utilization repressor PtxS	
1	894849	896030	1182	Phosphopentomutase (EC 5.4.2.7)	Deoxyribose and Deoxynucleoside Catabolism
1	896047	896877	831	Purine nucleoside phosphorylase (EC 2.4.2.1) @ N-Ribosylnicotinamide phosphorylase (EC 2.4.2.1)	
1	1270059	1270763	705	Purine nucleoside phosphorylase (EC 2.4.2.1)	
1	2295878	2296828	951	Deoxyribonucleoside regulator DeoR (transcriptional repressor)	
1	2297339	2298640	1302	Pyrimidine-nucleoside phosphorylase (EC 2.4.2.2)	
1	2298662	2299336	675	Deoxyribose-phosphate aldolase (EC 4.1.2.4)	
1	4307616	4306738	879	Ribokinase (EC 2.7.1.15)	
1	93533	94945	1413	6-phosphogluconate dehydrogenase, decarboxylating (EC 1.1.1.44)	D-gluconate and ketogluconates metabolism,
1	1808925	1809710	786	Glucose 1-dehydrogenase (EC 1.1.1.47)	
1	1894883	1895776	894	6-phosphogluconate dehydrogenase, decarboxylating (EC 1.1.1.44)	
1	2143936	2144895	960	2-ketogluconate kinase (EC 2.7.1.13)	
1	3866974	3866078	897	6-phosphogluconate dehydrogenase, decarboxylating (EC 1.1.1.44)	
1	4053713	4052355	1359	Gluconate transporter family protein	
1	4188763	4187978	786	Glucose 1-dehydrogenase (EC 1.1.1.47)	
1	4388192	4387407	786	Glucose 1-dehydrogenase (EC 1.1.1.47)	
1	4471081	4469753	1329	Gluconate permease	
1	4474697	4473156	1542	Gluconokinase (EC 2.7.1.12)	
1	4475622	4474723	900	6-phosphogluconate dehydrogenase, decarboxylating (EC 1.1.1.44)	
1	132344	132988	645	Transaldolase (EC 2.2.1.2)	Fructose utilization,

1	1591734	1592414	681	Transaldolase (EC 2.2.1.2)	
1	1895826	1896494	669	Transaldolase (EC 2.2.1.2)	
1	2790910	2790239	672	Transaldolase (EC 2.2.1.2)	
1	2986512	2985598	915	1-phosphofructokinase (EC 2.7.1.56)	
1	2987261	2986509	753	Transcriptional repressor of the fructose operon, DeoR family	
1	3033599	3032661	939	Fructokinase (EC 2.7.1.4)	
1	3267030	3266362	669	Transaldolase (EC 2.2.1.2)	
1	3895496	3893775	1722	Phosphoenolpyruvate-protein phosphotransferase of PTS system (EC 2.7.3.9)	
1	3895762	3895496	267	Phosphotransferase system, phosphocarrier protein HPr	
1	4043879	4042902	978	Fructokinase (EC 2.7.1.4)	
1	4307616	4306738	879	Ribokinase (EC 2.7.1.15)	D-ribose utilization,
1	4308598	4307609	990	Ribose operon repressor	
1	3051485	3049977	1509	Xylulose kinase (EC 2.7.1.17)	Xylose utilization
1	3254513	3253092	1422	D-xylose proton-symporter XylE	Xylose utilization
1	3256317	3254815	1503	Xylulose kinase (EC 2.7.1.17)	Xylose utilization
1	3257713	3256376	1338	Xylose isomerase (EC 5.3.1.5)	Xylose utilization
1	3257939	3259105	1167	Xylose-responsive transcription regulator, ROK family	Xylose utilization
1	1653329	1654960	1632	Ribulokinase (EC 2.7.1.16)	L-Arabinose utilization
1	1654954	1655652	699	L-ribulose-5-phosphate 4-epimerase (EC 5.1.3.4)	
1	1655682	1657106	1425	L-arabinose isomerase (EC 5.3.1.4)	
1	1657252	1658631	1380	Arabinose-proton symporter	

Adhesion					
**Scaffold**	**Start**	**Stop**	**Length (nt)**	**Function**	**Subsystems**
1	164583	165407	825	Flagellar basal-body rod protein FlgF	Flagellum
1	165431	166252	822	Flagellar basal-body rod protein FlgG	
1	191405	191872	468	Flagellar biosynthesis protein FlgN	
1	191891	193414	1524	Flagellar hook-associated protein FlgK	
1	193417	194298	882	Flagellar hook-associated protein FlgL	
1	205363	205764	402	Flagellar biosynthesis protein FliS	
1	250767	252077	1311	RNA polymerase sigma-54 factor RpoN	
1	485042	485845	804	Flagellar motor rotation protein MotA	
1	485880	486491	612	Flagellar motor rotation protein MotB	
1	761386	762519	1134	RNA polymerase sigma factor RpoD	
1	996121	998379	2259	Cell division protein FtsI [Peptidoglycan synthetase] (EC 2.4.1.129)	
1	998449	1000365	1917	Cell division protein FtsI [Peptidoglycan synthetase] (EC 2.4.1.129) @ Sporulation peptidoglycan synthetase SpoVD	
1	1090716	1091105	390	Flagellar basal-body rod protein FlgB	
1	1091108	1091557	450	Flagellar basal-body rod protein FlgC	
1	1091570	1091860	291	Flagellar hook-basal body complex protein FliE	

1	1091910	1093502	1593	Flagellar M-ring protein FliF
1	1093514	1094533	1020	Flagellar motor switch protein FliG
1	1094526	1095314	789	Flagellar assembly protein FliH
1	1095289	1096602	1314	Flagellum-specific ATP synthase FliI
1	1096605	1097048	444	Flagellar protein FliJ
1	1097058	1097690	633	Flagellar protein FlbB
1	1100345	1100773	429	Flagellar basal-body rod modification protein FlgD
1	1100799	1101593	795	Flagellar hook protein FlgE
1	1101623	1101838	216	Flagellar protein FlbD
1	1102294	1103295	1002	Flagellar motor switch protein FliM
1	1103285	1104415	1131	Flagellar motor switch protein FliN
1	1105497	1106165	669	Flagellar biosynthesis protein FliP
1	1106176	1106445	270	Flagellar biosynthesis protein FliQ
1	1106448	1107224	777	Flagellar biosynthesis protein FliR
1	1107227	1108309	1083	Flagellar biosynthesis protein FlhB
1	1108340	1110376	2037	Flagellar biosynthesis protein FlhA
1	1110376	1111473	1098	Flagellar biosynthesis protein FlhF
1	1111466	1112335	870	Flagellar synthesis regulator FleN
1	1116443	1117204	762	RNA polymerase sigma factor for flagellar operon
1	3087545	3086712	834	Flagellar motor rotation protein MotB

1	3088332	3087538	795	Flagellar motor rotation protein MotA	
1	4142047	4141232	816	Flagellin protein FlaA	
1	4324925	4326262	1338	RNA polymerase sigma-54 factor RpoN	
1	726275	725445	831	Late competence protein ComER, proline oxidase (EC 1.5.1.2)	- none -
1	726372	727007	636	Late competence protein ComEA, DNA receptor	- none -
1	727098	727664	567	dCMP deaminase (EC 3.5.4.12) @ Late competence protein ComEB	- none -
1	800238	801290	1053	Late competence protein ComGA, access of DNA to ComEA	- none -
1	801281	802327	1047	Late competence protein ComGB, access of DNA to ComEA	- none -
1	802342	802653	312	Late competence protein ComGC, access of DNA to ComEA, FIG007487	- none -
1	802640	803077	438	Late competence protein ComGD, access of DNA to ComEA, FIG012777	- none -
1	1036739	1035027	1713	Fibronectin/fibrinogen-binding protein	- none -
1	1316172	1317497	1326	Purple acid phosphatase/fibronectin domain protein	- none -
1	3339882	3341099	1218	Translation elongation factor Tu	Mycobacterium virulence operon involved in protein synthesis (SSU ribosomal proteins),<br>Translation elongation factors bacterial
1	5084846	5083656	1191	Translation elongation factor Tu	Mycobacterium virulence operon involved in protein synthesis (SSU ribosomal proteins),

					Translation elongation factors bacterial
1	4996262	4994631	1632	Heat shock protein 60 kDa family chaperone GroEL	- none -
1	4977120	4977797	678	Collagen triple helix repeat protein	- none -

Table S5. Comparison of assembly level, source, contig number, length, CDS number, rRNA number, and tRNA number of different *Lactococcus lactis* subsp. *lactis* in NCBI (https://www.ncbi.nlm.nih.gov/datasets/genomes/?taxon=1360&utm_source=gquery&utm_medium=referral).

Assembly Accession	Assembly Level	Source NCBI	Source Given	Contigs	Length	CDS	rRNA	tmRNA	tRNA
This study	Scaffold	Nile tilapia	Aquatic Animal	33	2501275	2395	7	1	53
GCF_000006865.1	Complete Genome	Not Given	Not Given	1	2365589	2383	19	1	61
GCF_000025045.1	Complete Genome	mung bean	Plant	2	2635654	2551	19	1	67
GCF_000192705.1	Complete Genome	Homo sapiens	Human	6	2518737	2495	19	1	63
GCF_000284735.1	Contig	Not Given	Not Given	131	2511332	2521	4	1	43
**GCF_000344575.1**	Complete Genome	Not Given	Not Given	1	2421471	2285	19	1	64
GCF_000348965.1	Contig	dairy	Food (Human)	71	2527313	2421	7	1	46
GCF_000442845.1	Contig	Not Given	Not Given	42	2700005	2692	9	1	55
GCF_000447905.1	Contig	propagated starter culture used in cheese manufacturing	Food (Human)	143	2505069	2549	9	1	59
GCF_000447985.1	Contig	propagated starter culture used in cheese manufacturing	Food (Human)	182	2550393	2561	10	1	58
GCA_000478255.2	Complete Genome	frozen peas	Food (Human)	2	2592264	2513	19	1	69
GCF_000479375.2	Complete Genome	home-made koumiss	Food (Human)	4	2595492	2610	19	1	61
GCF_000488975.1	Contig	starter culture for dairy fermentation	Food (Human)	132	2599239	2632	7	1	53
GCF_000493355.1	Scaffold	Not Given	Not Given	56	2603548	2622	5	1	52
**GCF_000703145.1**	Contig	Cow rumen	Terrestial animal	1	2480811	2469	19	1	66
GCF_000721865.3	Scaffold	Dromedary milk	Food (Human)	48	2338917	2269	1	1	40
GCF_000786755.1	Contig	artisanal cheese	Food (Human)	218	2576542	2549	3	1	21
GCF_000807375.1	Complete Genome	Fresh raw Milk	Food (Human)	1	2488699	2438	19	1	63
GCF_000835975.1	Contig	Not Given	Not Given	88	2545792	2568	4	1	52
**GCF_001437565.1**	Scaffold	Not Given	Not Given	45	2245449	2170	4	1	49
GCF_001455265.1	Contig	cheese	Food (Human)	83	2573719	2584	9	1	56
GCF_001456385.1	Contig	Dairy starter	Food (Human)	171	2547291	2503	10	1	53
**GCF_001456455.1**	Contig	Silage	Food (Animal)	46	2375664	2259	7	1	55
GCF_001456475.1	Contig	White kimchii	Food (Human)	60	2336037	2243	7	1	52
GCF_001456495.1	Contig	Alfalfa sprouts	Plant	54	2366759	2297	6	1	57
GCF_001456505.1	Contig	Alfalfa sprouts	Plant	192	2619218	2616	7	1	56
**GCF_001456525.1**	Contig	White kimchii	Food (Human)	61	2445522	2367	7	1	57
GCF_001456545.1	Contig	Grapefruit juice	Food (Human)	110	2684299	2620	6	1	58

GCF_001456575.1	Contig	Alfalfa and radish sprouts	Plant	44	2574519	2492	6	1	57
GCF_001456595.1	Contig	Japanese kaiwere shoots	Plant	38	2445893	2344	6	1	53
**GCF_001456615.1**	Contig	Mustard and cress	Plant	98	2651253	2557	7	1	52
**GCF_001456655.1**	Contig	Chinese radish seeds	Plant	48	2477489	2391	6	1	50
GCF_001456675.1	Contig	Frozen peas	Food (Human)	133	2488400	2427	6	1	58
GCF_001456685.1	Contig	Frozen peas	Food (Human)	227	2707537	2722	7	1	57
GCF_001456695.1	Contig	bovine milk	Food (Human)	225	2410930	2385	7	1	50
GCF_001456725.1	Contig	Alfalfa and radish sprouts	Plant	33	2463397	2360	6	1	61
**GCF_001456755.1**	Contig	Sliced mixed vegetables	Food (Human)	62	2376387	2281	6	1	52
GCF_001456775.1	Contig	Grass	Plant	75	2475932	2371	6	1	55
**GCF_001456785.1**	Contig	soil	Soil	149	2674319	2617	7	1	55
GCF_001456795.1	Contig	Dairy starter	Food (Human)	264	2521873	2480	6	1	51
GCF_001456835.1	Contig	Soil and grass	Soil	169	2743919	2691	6	1	54
GCF_001456855.1	Contig	Dairy starter	Food (Human)	213	2498421	2475	6	1	51
GCF_001456865.1	Contig	Dairy starter	Food (Human)	198	2473055	2423	6	1	53
GCF_001514475.1	Contig	Not Given	Not Given	40	2547623	2549	4	1	45
GCF_001622235.1	Contig	Dairy starter	Food (Human)	253	2457554	2414	7	1	52
GCF_001984785.1	Contig	starter culture	Food (Human)	41	2260841	2253	6	1	52
GCF_002078415.1	Complete Genome	Dairy fermentation	Food (Human)	6	2634132	2666	16	1	57
GCF_002078435.1	Complete Genome	Dairy fermentation	Food (Human)	5	2753892	2738	19	1	61
GCF_002078475.2	Complete Genome	Fermented Meat Product	Food (Human)	7	2538171	2434	19	1	63
GCF_002078495.1	Complete Genome	Dairy fermentation	Food (Human)	6	2542029	2539	19	1	61
GCF_002078615.1	Complete Genome	Dairy fermentation	Food (Human)	3	2607675	2636	22	1	69
GCF_002078855.1	Complete Genome	Dairy fermentation	Food (Human)	4	2459217	2475	18	1	60
GCF_002078975.2	Complete Genome	Dairy fermentation	Food (Human)	4	2680416	2613	19	1	67
GCF_002078995.2	Complete Genome	Fermented Meat Product	Food (Human)	6	2548507	2462	19	1	63
GCF_002148215.1	Complete Genome	10 weeks old Samso 45+ cheese	Food (Human)	8	2509249	2512	19	1	62
**GCF_002286195.1**	Complete Genome	marine fish gut	Aquatic Animal	3	2511408	2480	19	1	67
GCF_002441815.1	Contig	Not Given	Not GIven	76	2463410	2434	4	1	39
GCF_002563615.1	Scaffold	cheese	Food (Human)	151	2553575	2541	4	1	51
GCF_002804185.1	Complete Genome	Not Given	Not Given	1	2432058	2298	16	1	53
GCF_002804285.1	Complete Genome	Not Given	Not Given	1	2414663	2285	17	1	62

**GCF_002866835.1**	Contig	olive flounder	Aquatic Animal	3	2511408	2480	19	1	67
**GCF_002895225.1**	Complete Genome	Napier grass	Plant	1	2346663	2232	19	1	66
**GCF_003176835.1**	Complete Genome	almond drupe	Plant	2	2639081	2583	19	1	68
GCF_003594665.1	Contig	Not Given	Not Given	25	2439471	2371	5	1	52
GCF_003627395.2	Complete Genome	butter starter	Food (Human)	7	2702171	2758	19	1	63
GCF_003627415.1	Contig	butter starter	Food (Human)	4	2526633	2581	19	1	62
GCF_004354485.1	Scaffold	Not Given	Not Given	82	2627820	2600	25	1	64
GCF_005864305.1	Contig	goat milk	Food (Human)	49	2757319	2792	10	1	58
**GCF_006351065.1**	Contig	Green peas	Plant	41	2491705	2387	7	1	51
GCF_006538925.1	Contig	Not Given	Not Given	117	2436717	2421	4	1	38
GCF_008180075.1	Contig	3-day-old traditional semihard cheese made from mixed cow (20%) and sheep (80%) milk	Food (Human)	123	2655682	2643	7	1	51
GCF_008728875.1	Complete Genome	Not Given	Not Given	11	2636203	2628	19	1	62
GCF_009661795.1	Contig	Not Given	Not Given	113	2592713	2617	7	1	53
GCF_009661965.1	Contig	Not Given	Not Given	104	2392814	2385	7	1	51
GCF_009662035.1	Contig	Not Given	Not Given	140	2519952	2530	7	1	49
GCF_009662055.1	Contig	Not Given	Not Given	68	2298370	2306	7	1	50
GCF_009662075.1	Contig	Not Given	Not Given	104	2412036	2400	7	1	50
GCF_009662085.1	Contig	Not Given	Not Given	53	2259999	2251	7	1	51
GCF_009663915.1	Contig	dairy	Food (Human)	120	2445329	2440	4	1	52
GCF_009663925.1	Contig	dairy	Food (Human)	130	2552877	2558	5	1	52
GCF_009663935.1	Contig	Not Given	Not Given	132	2553867	2563	4	1	52
GCF_009885055.1	Scaffold	Not Given	Not Given	91	2424658	2387	4	1	52
GCF_013249195.1	Contig	Dairy product	Food (Human)	106	2589822	2562	7	1	52
GCF_013395015.3	Complete Genome	farm kitchen	Environment	5	2706818	2790	65	19	1
GCF_015714805.1	Contig	Ethiopian Ergo	Food (Human)	148	2669227	2636	4	1	49
GCF_016921015.1	Complete Genome	Dairy Fermentation	Food (Human)	7	2530852	2553	19	1	63
GCF_016952955.1	Complete Genome	Dairy Fermentation	Food (Human)	6	2611387	2650	19	1	63
GCF_017821195.1	Complete Genome	Dairy	Food (Human)	8	2618227	2639	16	1	59
GCF_017869415.1	Complete Genome	dairy	Food (Human)	1	2409036	2377	19	1	62
GCF_018195735.1	Contig	milk camel	Food (Human)	34	2513815	2445	7	1	55
GCF_018195745.1	Contig	milk camel	Food (Human)	118	2872551	2874	7	1	53
GCF_018195775.1	Contig	milk camel	Food (Human)	36	2514212	2444	7	1	55

GCF_018195795.1	Contig	milk camel	Food (Human)	115	2815350	2816	7	1	53
GCF_018195805.1	Contig	milk camel	Food (Human)	38	2513704	2443	7	1	55
GCF_018195835.1	Contig	milk camel	Food (Human)	114	2814330	2816	7	1	53
GCF_018320725.1	Contig	fermented buffalo milk, Dadih	Food (Human)	55	2441808	2383	4	1	48
GCF_900088425.1	Complete Genome	Not Given	Not Given	5	2730625	2658	19	1	64
GCF_900291885.1	Contig	Not Given	Not Given	78	2613255	2633	12	1	66
GCF_900291895.1	Contig	Not Given	Not Given	78	2575082	2582	8	1	54

Table S6. Comparison of assembly level, source, contig number, length, CDS number, rRNA number, and tRNA number of different *Priestia megaterium* genomes in NCBI. (https://www.ncbi.nlm.nih.gov/datasets/genomes/?taxon=1404).

**Assembly Accession**	**Assembly Level**	**Source**	**Source given**	**Contig**	**Length**	**CDS**	**rRNA**	**tmRNA**	**tRNA**
This study	Scaffold	Nile tilapia	Aquatic animal	94	5485864	5576	23	1	142
GCF_000025805.1	Complete Genome	Not Given	Not Given	1	5097447	5191	33	1	115
GCF_000025825.1	Complete Genome	Not Given	Not Given	8	5523192	5596	37	1	137
GCF_000225265.1	Complete Genome	Not Given	Not Given	4	5075293	5209	33	1	117
GCF_000334875.3	Complete Genome	contaminated soil	Environmental	11	5883957	5955	53	1	140
GCF_000480335.1	Scaffold	salt crystallizer of the Rann of Kutch	Salt	140	4369029	4116	4	1	56
GCF_000527015.1	Contig	Not Given	Not Given	47	5460379	5572	6	1	62
GCF_000746935.1	Scaffold	Not Given	Not Given	5	5620959	5742	21	1	111
GCF_000764085.1	Contig	Not Given	Not Given	78	5727066	5866	18	1	58
GCF_000832985.1	Complete Genome	environmental sample	Environmental	7	5746640	5858	41	1	122
GCF_000876465.1	Contig	submerged soil sample, rice field	Environmental	49	5543117	5684	13	1	94
GCF_001029885.1	Contig	Chamaecostus cuspidatus internal stem tissue	Plant	240	5864214	5946	21	1	148
GCF_001050455.1	Complete Genome	Not Given	Not Given	2	5229799	5264	42	1	130
**GCF_001238505.1**	Contig	Soil	Environmental	194	5669134	5750	4	1	98
**GCF_001541965.1**	Contig	Waltherarndtia caliculatum White Sands Reef	Aquatic Animal	167	5435297	5521	15	1	144
GCF_001591525.1	Contig	Not Given	Not Given	50	5513953	5663	10	1	84
GCF_001593805.1	Scaffold	bar-headed goose	Bird	78	6247384	6413	5	1	116
GCF_001640745.1	Contig	human gut microbiota	Human	12	5051644	5213	16	1	107
GCF_001937775.1	Contig	lake water	Environmental	87	5974410	6087	26	1	151
GCF_002009195.1	Complete Genome	Camellia oleifera	Plant	8	5610875	5816	44	1	119
GCF_002276335.1	Contig	uncut heroin sample	Drug	348	5781865	5825	3	1	28
GCF_002550995.1	Scaffold	soil	Environmental	151	6082026	6185	6	1	77
GCF_002551875.1	Scaffold	soil	Environmental	138	6018513	6158	4	1	69
GCF_002552135.1	Scaffold	root Soybean	Plant	47	5439139	5564	4	1	94
GCF_002552565.1	Contig	Wheat	Plant	51	5321764	5467	3	1	88
GCF_002552835.1	Scaffold	soil	Environmental	161	6092226	6214	5	1	64
GCF_002552955.1	Scaffold	soil	Environmental	136	5990546	6124	4	1	77
GCF_002557935.1	Scaffold	root Corn	Plant	121	5898346	6068	3	1	72

**GCF_002558155.1**	Contig	Grass	Plant	64	5315331	5420	4	1	96
GCF_002558805.1	Scaffold	root Corn	Plant	127	5973872	6111	4	1	82
GCF_002559285.1	Scaffold	root Corn	Plant	76	5752868	5886	5	1	93
GCF_002559335.1	Scaffold	soil	Environmental	58	5824724	5972	4	1	84
GCF_002559475.1	Scaffold	root Soybean	Plant	55	5891653	6013	4	1	86
**GCF_002560595.1**	Scaffold	Corn	Plant	36	5130959	5179	4	1	79
GCF_002560705.1	Scaffold	root Corn	Plant	102	5873408	5970	3	1	78
**GCF_002561015.1**	Scaffold	root Corn	Plant	43	5430055	5529	4	1	86
GCF_002561125.1	Scaffold	Grass	Plant	75	5933109	6101	4	1	73
GCF_002561515.1	Scaffold	root Cotton	Plant	118	5630529	5740	4	1	79
GCF_002562195.1	Scaffold	Grass	Plant	54	5736173	5877	4	1	64
GCF_002562515.1	Scaffold	soil	Environmental	82	5757646	5876	5	1	77
**GCF_002564705.1**	Scaffold	root Corn	Plant	60	5426973	5508	3	1	78
GCF_002564755.1	Scaffold	soil	Environmental	166	6050940	6236	4	1	86
GCF_002565155.1	Scaffold	root Corn	Plant	76	5858387	5975	3	1	81
GCF_002565335.1	Scaffold	soil	Environmental	104	6003007	6107	4	1	81
GCF_002565535.1	Scaffold	root Soybean	Plant	41	5688351	5812	4	1	78
GCF_002566025.1	Scaffold	soil	Environmental	95	5940000	6055	4	1	82
GCF_002566035.1	Scaffold	Corn	Plant	250	6065517	6189	5	1	56
GCF_002566285.1	Scaffold	root Corn	Plant	136	6022330	6127	4	1	69
**GCF_002566345.1**	Scaffold	root Corn	Plant	55	5550488	5679	6	1	97
GCF_002566435.1	Scaffold	root Soybean	Plant	53	5815091	5970	4	1	68
GCF_002566965.1	Scaffold	vegetable core	Plant	98	5915645	6046	5	1	90
GCF_002566985.1	Scaffold	root Corn	Plant	74	6060134	6208	3	1	63
GCF_002567045.1	Scaffold	core Grass	Plant	92	5784293	5938	4	1	75
GCF_002567075.1	Scaffold	soil	Environmental	124	5862733	6035	3	1	81
**GCF_002567645.1**	Scaffold	plant root	Plant	86	5325730	5422	4	1	91
GCF_002568195.1	Scaffold	plant root	Plant	83	5361395	5525	4	1	80
GCF_002568305.1	Scaffold	root Corn	Plant	58	5851041	5955	3	1	70
GCF_002570645.1	Scaffold	root Corn	Plant	60	5854016	5963	4	1	81
**GCF_002570715.1**	Scaffold	core Corn	Plant	60	5429875	5541	4	1	93
GCF_002571125.1	Scaffold	root Corn	Plant	80	5968560	6083	4	1	85

GCF_002574105.1	Scaffold	root Corn	Plant	54	5846869	6005	4	1	88
**GCF_002574795.1**	Scaffold	plant root	Plant	78	5502575	5561	5	1	88
GCF_002574835.1	Scaffold	soil	Environmental	76	7057766	7417	4	1	91
GCF_002575285.1	Scaffold	vegetable core	Environmental	88	5888189	6016	3	1	79
GCF_002575465.1	Scaffold	root Corn	Plant	81	6114345	6270	5	1	73
GCF_002575685.1	Scaffold	root Corn	Plant	42	5643538	5781	4	1	73
GCF_002575735.1	Scaffold	soil	Environmental	112	6099229	6298	3	1	86
GCF_002576235.1	Scaffold	core Corn	Plant	100	6002747	6164	3	1	85
GCF_002576315.1	Scaffold	core Grass	Plant	72	5818223	5967	4	1	76
GCF_002576505.1	Scaffold	root Soybean	Plant	67	6017010	6104	3	1	75
**GCF_002576935.1**	Scaffold	soil	Environmental	150	5127880	5245	4	1	79
GCF_002577015.1	Scaffold	core Corn	Plant	109	6060373	6201	5	1	84
GCF_002577035.1	Scaffold	soil	Environmental	66	5784810	5918	4	1	82
GCF_002577555.1	Scaffold	core Grass	Plant	96	6067869	6176	4	1	63
GCF_002577645.1	Scaffold	core Corn	Plant	68	5973353	6077	4	1	103
GCF_002583135.1	Scaffold	soil	Environmental	106	5926154	6028	4	1	78
GCF_002584405.1	Scaffold	root Corn	Plant	91	5955022	6066	4	1	87
GCF_002585805.1	Scaffold	root Corn	Plant	66	5872152	5986	3	1	77
**GCF_002586045.1**	Scaffold	Soil	Environmental	39	5190353	5284	3	1	72
GCF_002586445.1	Scaffold	Soil	Environmental	129	5932001	6041	3	1	71
**GCF_002871835.1**	Scaffold	Homo sapiens	Human	16	5254734	5319	12	1	74
GCA_002872495.3	Chromosome	Soil	Environmental	2	5121099	5237	10	1	73
**GCF_002888675.1**	Contig	stored rice grains	Plant	98	5022643	5110	19	1	100
GCF_003072605.2	Complete Genome	Rhizosphere Sporobolus anglicus	Plant	5	5432127	5430	42	1	130
GCF_003074515.1	Contig	Rhizosphere Sporobolus anglicus	Plant	36	5475756	5570	54	1	164
GCF_003075235.1	Contig	Bioreactor	Not Given	16	5071852	5165	28	1	118
GCF_003075275.1	Contig	Bioreactor	Not Given	32	5081950	5183	25	1	100
GCF_003075295.1	Contig	Bioreactor	Not Given	28	5084955	5175	32	1	121
GCF_003075355.1	Contig	Bioreactor	Not Given	69	5089878	5191	22	1	104
GCF_003382345.1	Scaffold	Soil	Environmental	114	5920616	6125	9	1	87
GCF_003688675.1	Scaffold	Not Given	Not Given	13	4937440	5023	10	1	72

GCF_004150295.1	Contig	Clay soil high temperatures around 45 celsius degrees	Environmental	247	5572365	5634	18	1	132
GCF_004803605.1	Contig	fermented food	Food (Human)	101	5596120	5719	8	1	82
**GCF_005048835.1**	Contig	Soil	Environmental	47	5501316	5598	13	1	97
GCF_005491525.1	Chromosome	Air	Environmental	8	5497494	5587	34	1	130
GCF_006094495.1	Complete Genome	Not Given	Not Given	7	5746548	5851	41	1	120
GCF_006385935.1	Complete Genome	Soil	Environmental	6	5712573	5808	43	1	137
GCF_006454485.1	Scaffold	silkworm excrement	Insect	29	5319455	5521	1	1	44
GCF_006455245.1	Scaffold	silkworm excrement	Insect	36	5323602	5524	1	1	48
**GCF_006716955.1**	Scaffold	Not Given	Not Given	16	5388835	5517	11	1	90
GCF_007672525.1	Scaffold	environmental sample	Environmental	626	5520005	5604	4	1	50
GCF_007672925.1	Scaffold	environmental sample	Environmental	62	5271307	5378	4	1	50
GCF_007673295.1	Scaffold	environmental sample	Environmental	1008	5503411	5516	4	1	46
GCF_007674025.1	Scaffold	environmental sample	Environmental	136	5418958	5537	5	1	47
GCF_007674145.1	Scaffold	environmental sample	Environmental	88	5649004	5735	7	1	46
GCF_007677165.1	Scaffold	environmental sample	Environmental	110	5248653	5369	4	1	40
GCF_007678145.1	Scaffold	environmental sample	Environmental	63	5364424	5535	5	1	82
GCF_007678435.1	Scaffold	environmental sample	Environmental	119	5787672	5906	6	1	50
GCF_007923065.1	Chromosome	sludge	Environmental	1	5072144	5162	11	1	102
GCF_007923105.1	Chromosome	sludge	Environmental	1	6169081	6313	9	1	106
GCF_008693225.1	Scaffold	horsechestnut trunk	Plant	105	5133407	5246	5	1	71
GCF_009497655.1	Complete Genome	plant	Plant	3	5243446	5303	27	1	119
GCF_009728545.1	Contig	Retama monosperma root nodules	Plant	81	5817483	5818	21	1	118
GCF_009911775.1	Complete Genome	farmland	Environmental	1	5171845	5242	40	1	118
GCF_009935415.1	Scaffold	Commercial Probiotic	Not Given	28	5600120	5721	3	1	65
GCF_010178635.1	Contig	spacecraft hardware	Other	12	5761571	5940	49	1	147
GCF_011058275.1	Complete Genome	soil	Environmental	2	5407472	5486	37	1	118
GCF_012275205.1	Complete Genome	Not Given	Not Given	1	6468885	6177	63	1	149
GCF_012641205.1	Contig	Sapindus	Plant	62	5102251	5195	15	1	95
GCF_012926555.1	Contig	Not Given	Not Given	196	5429269	5535	19	1	105
GCF_013146705.1	Complete Genome	Paper surface	Environmental	10	6872701	7089	47	1	125
GCF_013375615.1	Contig	Zea mays	Plant	104	6160258	6323	1	1	61
GCF_013389435.1	Complete Genome	potash salt dump	Environmental	10	5984922	6061	47	1	153

GCF_013458535.1	Complete Genome	agricultural soil	Environmental	10	6165405	6231	47	1	158
GCF_014839485.1	Contig	Phaseolus vulgaris seed	Plant	32	5775775	5892	17	1	126
GCF_014842705.1	Contig	Phaseolus vulgaris seed	Plant	57	6069910	6202	18	1	121
GCF_014892475.1	Scaffold	soil sample (organo-mineral particles) near a borehole	Environmental	122	6183957	6371	4	1	53
GCF_015354485.1	Contig	Endophyte	Plant	54	6086124	6206	19	1	152
**GCF_015582655.1**	Complete Genome	Water	Environmental	1	5373548	5508	10	1	119
GCF_015643545.1	Complete Genome	bulk soil of maize	Environmental	1	5147837	5219	39	1	112
GCF_015880925.1	Contig	maybe soil	Environmental	42	5509502	5665	16	1	92
GCF_016892545.1	Contig	Grass field Lolium arundinaceum	Plant	113	5749343	5905	5	1	85
GCF_017086525.1	Complete Genome	Not Given	Not Given	8	5747602	5853	41	1	122
GCF_017086545.1	Complete Genome	Homo sapiens	Human	21	5608820	5629	45	1	147
GCF_017086565.1	Complete Genome	Homo sapiens	Human	14	5999955	6050	46	1	139
GCF_017352315.1	Complete Genome	Hypersaline environment	Environmental	19	6412227	6581	47	1	147
GCF_017798265.1	Complete Genome	mulberry	Plant	4	5268816	5324	42	1	130
GCF_018101235.1	Contig	roots Brassica napus	Plant	97	5991912	6140	16	1	108
GCF_018615005.1	Scaffold	soil	Environmental	77	5646934	5888	7	0	60
GCF_018615055.1	Scaffold	soil	Environmental	71	6068635	6293	9	1	77
GCF_019037025.1	Scaffold	cleanroom floor	Environmental	52	5855780	5975	6	1	48
GCF_019037625.1	Scaffold	cleanroom floor	Environmental	67	6163057	6319	5	1	48
GCF_019038045.1	Scaffold	cleanroom floor	Environmental	69	5616472	5748	5	1	47
GCF_019193015.1	Contig	Poa pratensis L.	Plant	141	6167380	6245	15	1	122
GCF_019281975.1	Scaffold	Homo sapiens	Human	176	6169273	6269	10	1	78
GCA_019641605.1	Contig	farm soil	Environmental	61	5269082	5358	14	1	88
GCF_019748595.1	Contig	Air filter debris	Environmental	98	5930230	6068	8	1	111
GCA_020176875.1	Contig	roots coffee	Plant	10	5234742	5249	46	1	152
**GCF_020179075.1**	Contig	rizosphere wheat	Plant	34	5271792	5389	17	1	119
GCF_020251185.1	Contig	Soil	Environmental	67	6004406	6179	9	1	72
GCF_900113355.1	Scaffold	Not Given	Not Given	81	5886428	6043	9	1	77
GCF_900445445.1	Contig	environmental	Environmental	4	5364371	5388	39	1	131
GCF_900445485.1	Contig	Not Given	Not Given	8	5782255	5919	41	1	122

Table S7. Comparison of the assembly level, source, number of contigs, length, number of CDS, number of rRNA and number of tRNA from different genomes of *Priestia aryabhattai* in NCBI (https://www.ncbi.nlm.nih.gov/datasets/genomes/?taxon=412384).

Assembly Accession	Assembly Level	Source (NCBI)	Source Given	Contig	Length	CDS	rRNA	tmRNA	tRNA
This study	Scaffold	Nile Tilapia	Aquatic Animal	42	5337734	5443	27	1	124
GCF_000785045.1	Contig	deep-sea water	Environment	37	5105129	5212	6	1	79
GCF_000956595.1	Contig	high-altitude air sample	Environment	72	5095483	5210	5	1	78
GCF_001038965.1	Contig	Plant	Plant	48	5421362	5628	12	1	111
GCF_001043825.1	Contig	Plant	Plant	109	5499219	5683	11	1	117
GCF_001619595.1	Contig	Ulu Slim Hot Spring	Environment	37	4963060	5092	9	1	86
GCF_001858395.1	Scaffold	Rhizospheric soil	Environment	23	5403026	5487	11	1	118
GCF_002167185.1	Contig	lake sediment	Environment	352	4599278	4654	13	1	110
GCF_002208605.1	Contig	date palm	Plant	118	5584517	5690	5	1	73
GCF_002265635.1	Contig	Domestic sewerage soil	Environment	72	4193880	4265	10	1	82
GCF_002555815.1	Scaffold	root	Plant	83	5300455	5457	2	1	78
GCF_002569015.1	Scaffold	Wheat	Plant	87	5410757	5552	5	1	88
GCF_002569785.1	Scaffold	soil	Environment	53	5100887	5218	2	1	72
GCF_002588685.1	Scaffold	soil	Environment	300	5381867	5438	4	1	89
GCF_002688605.1	Complete Genome	enriched culture of compost	Environment	3	5254250	5310	42	1	130
GCF_013372535.1	Contig	soil	Environment	34	5172894	5236	13	1	80
GCF_014138775.1	Scaffold	Not Given	Not Given	48	6054250	6212	10	1	80
GCF_014932885.1	Scaffold	clay soil	Environment	82	6169737	6336	5	1	54
GCF_015845475.1	Contig	Not Given	Not Given	35	5497028	5592	22	1	131
GCF_017743055.1	Complete Genome	soil	Environment	6	5956834	6039	43	1	142
GCF_018881345.1	Contig	tobacco seed	Plant	6	5815073	5848	43	1	137
GCF_019748635.1	Contig	Air filter	Environment	87	5462892	5602	11	1	119
GCF_019748735.1	Contig	Air filter	Environment	62	5354727	5491	6	1	113
GCF_019748795.1	Contig	Air filter	Environment	33	5509225	5591	9	1	96
GCF_019748815.1	Contig	Air filter	Environment	46	5210447	5278	8	1	100
GCF_019748835.1	Contig	Air filter	Environment	54	5127833	5215	7	1	99
GCF_019748845.1	Contig	Air filter	Environment	47	5100431	5184	6	1	96

GCF_019748955.1	Contig	Air filter	Environment	38	5177591	5286	5	1	87
GCF_019748975.1	Contig	Air filter	Environment	53	5104184	5182	5	1	100
GCF_019748995.1	Contig	Air filter	Environment	36	5156817	5222	9	1	91
GCA_019749195.1	Contig	HEPA air filter (JPL lab)	Environment	119	6016092	6135	11	1	118
GCF_019749215.1	Contig	HEPA air filter (JPL lab)	Environment	76	5662423	5761	9	1	102
GCA_019749225.1	Contig	HEPA air filter (JPL lab)	Environment	99	6011509	6137	14	1	111
GCF_019968665.1	Complete Genome	mangrove sediment	Environment	4	5257678	5271	42	1	130
GCF_020102825.1	Scaffold	Microcosmus	Aquatic Animal	33	5640273	5684	9	1	81
GCF_020171825.1	Contig	Mangrove rhizosphere sediment	Environment	22	5144759	5241	12	1	107
GCF_020524445.1	Contig	Mangrove rhizosphere sediment	Environment	51	5425686	5596	14	1	112
GCF_020524855.1	Contig	Mangrove rhizosphere sediment	Environment	32	5158491	5248	13	1	96
GCF_903971025.1	Contig	Pterocarpus santalinus leaf	Plant	160	5632564	5830	30	1	129
GCF_903971035.1	Contig	Pterocarpus santalinus leaf	Plant	120	5475026	5711	28	1	99
